# Continuous Monitoring of the Thermoregulatory Response in Endurance Horses and Trotter Horses During Field Exercise: Baselining for Future Hot Weather Studies

**DOI:** 10.3389/fphys.2021.708737

**Published:** 2021-08-26

**Authors:** Elisabeth-Lidwien J. M. M. Verdegaal, Gordon S. Howarth, Todd J. McWhorter, Berit Boshuizen, Samantha H. Franklin, Carmen Vidal Moreno de Vega, Stacey E. Jonas, Louise E. Folwell, Catherine J. G. Delesalle

**Affiliations:** ^1^Equine Health and Performance Centre, University of Adelaide, Adelaide, SA, Australia; ^2^School of Animal and Veterinary Sciences, Roseworthy Campus, University of Adelaide, Adelaide, SA, Australia; ^3^Research Group of Comparative Physiology, Department of Virology, Parasitology and Immunology, Faculty of Veterinary Medicine, Ghent University, Ghent, Belgium

**Keywords:** thermoregulation, hyperthermia, gastrointestinal pill, endurance, trotters, metabolic heat (H), recovery, exercise

## Abstract

Establishing proper policies regarding the recognition and prevention of equine heat stress becomes increasingly important, especially in the face of global warming. To assist this, a detailed view of the variability of equine thermoregulation during field exercise and recovery is essential. 13 endurance horses and 12 trotter horses were equipped with continuous monitoring devices [gastrointestinal (GI) pill, heartrate (HR) monitor, and global positioning system] and monitored under cool weather conditions during four endurance rides over a total of 80 km (40 km loops) and intense trotter track-based exercise over 1,540 m. Recordings included GI temperature (T_*c*_), speed, HR and pre- and post-exercise blood values. A temperature time profile curve of T_*c*_ was constructed, and a net area under the curve was calculated using the trapezoidal method. Metabolic heat production and oxygen cost of transport were also calculated in endurance horses. Maximum T_*c*_ was compared using an independent samples *t*-test. Endurance horses (mean speed 14.1 ± 1.7 km h^–1^) reached mean maximum T_*c*_ (39.0 ± 0.4°C; 2 × 40 km in 8 horses) during exercise at 75% of completion of T_*c*_ exercise and T_*c*_ returned to baseline within 60 min into recovery. However, the mean T_*c*_ was still 38.8 ± 0.4°C at a HR of 60 bpm which currently governs “fit to continue” competition decisions. Trotters (40.0 ± 2.9 km h^–1^) reached a comparable mean max T_*c*_ (38.8 ± 0.5°C; 12 horses) always during recovery. In 30% of trotters, T_*c*_ was still >39°C at the end of recovery (40 ± 32 min). The study shows that horses are individuals and thermoregulation monitoring should reflect this, no matter what type of exercise is performed. Caution is advised when using HR cut-off values to monitor thermal welfare in horses since we have demonstrated how T_*c*_ can peak quite some time after finishing exercise. These findings have implications for training and management of performance horses to safeguard equine welfare and to maximize performance.

## Introduction

Exertional heat illness (EHI) in horses is characterized by severe central nervous system dysfunction, such as physical collapse, and has been described in detail (Brownlow et al., 2016; Brownlow and Mizzi, 2020). Recently, a Japanese study identified a wet-bulb globe temperature (WBGT) index above 28°C to be responsible for a 28.5% higher risk for development of EHI when compared to the index below 20°C (Takahashi and Takahashi, 2020). A progressive increase in the prevalence of human EHI casualties is anticipated due to global warming (Raymond et al., 2020) and a similar effect can be anticipated for animals in general. In continents with warm climates and established horseracing industries, a WBGT above 28°C is often recorded during approximately 2–3 months annually. These regions include the southern part of North America and Europe, Oceania, parts of Africa, South America, and Asia (Raymond et al., 2020). It is a common misconception that EHI affects horses in only very hot and humid weather conditions, however, EHI casualties are also reported to occur on warmer pre-season days. To date, EHI has been underreported most probably because only the more severe and overt clinical cases are being recognized, leaving many mild cases unnoticed with possibly deleterious effects (Brownlow et al., 2016; Brownlow and Mizzi, 2021).

Consequently, there is great need to further fine-tune early detection of hyperthermia and to obtain a detailed view of the flexibility of equine thermoregulation in the field under different exercise and climatic conditions. Such knowledge will allow for identification of predisposing factors that may lead to the formulation of evidence-based hot weather policies and recommendations for the post-exercise and recovery period to prevent hyperthermia, EHI and EHS (Hosokawa et al., 2019; MOOC heat illness, 2021). Another critical aspect that needs to be clarified is the optimal rest and recovery period that is required before “return to exercise” is allowed after an EHI event. For example, human athletes who have experienced an EHI episode are required to rest and rehabilitate for a certain period, varying from 3 to 6 weeks to a year (Adams and Jardine, 2020). Similarly, various regulations govern eliminated endurance horses who are given rest periods to recover following a competition (AERA, 2020; FEI, 2021).

The way the thermoregulatory system is challenged greatly depends on the speed and distance that are realized by the exercising horse (Hodgson et al., 1994). Additionally, important breed differences are to be expected (Fielding et al., 2011). For example, trotter horses are bred for harness racing over distances ranging from 1,540 m (short distance races), 2,140 m (medium distance races), 2,640 m (long distance races), and rarely to 3,140 m (ultra-long-distance races), with average race speeds of 47.3–48.6 km h^–1^ (Bertuglia et al., 2014). In comparison, endurance horses compete over distances of 80–160 km divided over exercise loops (sections) of 30–40 km under the regulations of either the National Endurance Riding Associations or the Fédération Équestre Internationale (FEI, 2018; FEI, 2021). The range of mean winning speeds at FEI 100–120 km endurance rides around the world varies between 14.1 and 24.8 km h^–1^ while reported maximum winning speeds range from 17.2 to 26.2 km h^–1^ (Nagy et al., 2010). After each exercise loop, a recovery rest period is imposed to allow the HR to return to below values of 60 bpm under the regulations of the Australian Endurance Riding Association or 64 bpm as per FEI regulations (Nagy et al., 2012; FEI, 2018; AERA, 2020). Once this is achieved, a follow-up inspection including metabolic and gait assessment is performed by the certified endurance veterinarian to determine whether the horse is deemed fit to continue with the next exercise loop or qualifies for completion of the competition. Clinical assessment during endurance rides is essential to ensure sufficient recovery in all horses since a high elimination rate (nearly 50%) is reported, and more specifically, the elimination rate due to metabolic disorders in endurance exercise varies between 4.2 and 15% (Barnes et al., 2010; Nagy et al., 2010, 2014; Fielding et al., 2011, 2017; Younes et al., 2015; Bennet and Parkin, 2018; Legg et al., 2019). Common debilitating metabolic disorders include abdominal discomfort, dehydration, and exertional rhabdomyolysis (Fielding et al., 2009; Verdegaal et al., 2018; Misheff, 2020).

At present, little is known about the core body temperature evolvement in real time during different types of exercise performed by racehorses and endurance horses under field conditions. The vast majority of thermoregulatory studies have been conducted under indoor laboratory conditions using a treadmill and subjecting the horses to performance of specific standardized exercise tests. To this end, monitoring of arterial pulmonary blood temperature has proven to be “the gold standard” (Hodgson et al., 1993; Marlin et al., 1996; Courouce-Malblanc, 2013; Hodgson, 2014). However, this approach is generally not feasible and too invasive under field conditions; only one study reported similar invasive methods in three horses during free field exercise using thermistors to measure blood and brain temperature (Mitchell et al., 2006). A few studies are available that focus on continuous monitoring of equine thermoregulation during exercise and recovery in the field (Smith et al., 2006; Verdegaal et al., 2017). Smith et al. (2006) used intra-uterine temperature loggers as a non-surgical, minimally invasive method to measure evolvement of intra-uterine temperature during field exercise. This method has the advantage of collecting data over several competitions, however, it does not monitor real-time core body temperature as data were downloaded after removal of the logger. Obviously, the intra-uterine method is only applicable in mares. No data are available on the long-term effect of these loggers on uterine function. In contrast, our telemetric recording method is able to provide real-time results and does not require subsequent time-critical analysis. This could be undertaken at any time after completion of the exercise period (Verdegaal et al., 2017).

It is important to note that most existing field studies compare rectal temperature (T_*re*_) before and after exercise (Kohn and Hinchcliff, 1995; Kohn et al., 1995; Hargreaves et al., 1999; Jeffcott et al., 2009; Wallsten et al., 2012). However, T_*re*_ is reported to significantly lag behind the core body temperature both during and after exercise (Hodgson et al., 1993; Geor et al., 1995; Verdegaal et al., 2017). This means that the heating of the core of the body is always reflected in the T_*re*_ at a much later stage and at a lower temperature. Recently, we reported and validated a novel real-time temperature monitoring method namely a gastrointestinal (GI) pill that allows for accurate continuous recording of the GI temperature in exercising horses in the field (Verdegaal et al., 2017). The GI pill proved to be a more accurate and precise tool to monitor thermal response than serial T_*re*_ measurements. Importantly, the GI temperature (T_*c*_) was consistently and significantly higher than the T_*re*_ (mean difference 0.3°C, with a range of 0.2–0.3°C) and T_*c*_ increased earlier than T_*re*_ on all occasions (Verdegaal et al., 2017).

The aim of the current study was to continuously monitor and compare the time profile of the dynamic thermoregulatory response of endurance horses in real-time competitions and trotter horses in the field. The GI temperature profiles were established during exercise and recovery under cool weather conditions to create a baseline thermal response profile for future field studies performed under hot and humid weather conditions. For this purpose, trotter and endurance horses were equipped with several non-invasive monitoring devices [GI pill (T_*c*_), heartrate (HR) monitor, and global positioning system (GPS) monitor] to continuously monitor their physiologic responses to field exercise.

## Materials and Methods

### Horses

**FIGURE 1:**
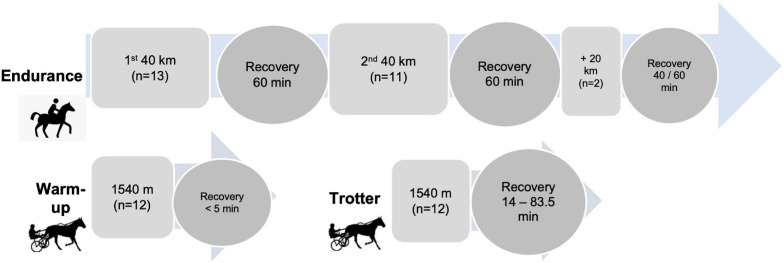
Flow chart of study design describing two types of exercise and distance; km, kilometers; m, meters; n, number of horses; min, minutes; endurance exercise; moderate trotter warm-up exercise; and fast trotter exercise.

In the first study group, 13 trained endurance horses (Horses 1–13) were enrolled, competing at either 40, 80, or 100 km distances. The study design is presented in a flowchart in Figure 1. The characteristics of the horses such as breed, age, sex, body mass, coat color, together with competition distance are presented in Table 1. The body mass of endurance horses was determined using an equine mass tape (Horse and pony weight tape^®^; Wagner and Tyler, 2011). When calculating the energy expenditure of the horses, the estimated mass of the rider was considered (Pagan and Hintz, 1986) based on “rider riding division criteria” determined in the AERA rules (AERA, 2020). These criteria encompass: (1) HM division—heavy mass rider of 91 kg or greater, estimated average 103 kg; (2) MM division—middle mass of 73 kg or greater, estimated average 83 kg; (3) LM division—low mass, no minimum mass, estimated average 63 kg. The trotter horses were weighed on a mass scale (Ruddweigh 700^®^, Gallagher Group Limited, New Zealand).

**TABLE 1:** Study population characteristics and its monitoring devices.

Horse number	Sex	Age (y)	Breed	Body mass (kg)	Total mass incl rider	Coat color	Distance (km)	Horse experience (years)	Age start (years)	GI Pill Y/N	GPS/HR Y/N	BOM (°C; T_*a*_), min – max	PCV (Hct), lactate, TS, pH Y/N	Blood gas Y/N	Biochemistry (electrolytes, glucose) Y/N	Hematology
1	G	11	Arab	669	752	Gr	80	6	4	Y	Y	13−26	Y	Y	Y	N
2	G	9	Arab	484	587	B	80	2	7	Y	Y	13−26	Y	Y	Y	N
3	M	13	Arab	426	489	C	80	3	10	Y	Y	6−19	Y	Y	Y	N
4	M	7	QH × TB	470	573	C	100	1	6	Y	Y	6−19	Y	Y	Y	N
5	M	11	Arab	450	533	C	80	2	9	Y	Y	6−19	Y	Y	Y	N
6	M	8	Arab	370	453	Gr	100	4	4	Y	Y	6−19	Y	Y	Y	N
7	M	9	Arab	450	523	C	80	2	7	Y	N^	3−22	Y	N	Y**	Y
8	G	11	Arab	470	553	Gr	80	1	9	Y	Y	3−22	Y	N	Y**	Y
9	G	7	QH	490	573	C	80	1	6	N	Y	7−13	Y	Y	Y	N
10	G	10	Arab × TB	484	587	C	80	5	5	N	Y	7−13	Y	Y	Y	N
11	G	5	Arab	458	541	B	40	0	5	N	Y	7−13	Y	Y	Y	N
12	G	7	Arab	525	608	B	80	NK	NK	N	Y	3−22	Y	N	Y**	Y
13	M	15	Arab	480	563	Gr	40	NK	NK	N	N^^	3−22	Y	N	Y**	Y
14	G	10	SB	484	−	B	1.54	−	−	Y	Y	5−13	Y	N	N	N
15	G	5	SB	610	−	B	1.54	−	−	Y	Y	5−13	Y	N	N	N
16	G	17	SB	482	−	B	1.54	−	−	Y	Y	5−13	Y	N	N	N
17	G	5	SB	604	−	B	1.54	−	−	Y	Y	5−13	Y	N	N	N
18	G	7	SB	554	−	B	1.54	−	−	Y	Y	3−18	Y	N	N	N
19	G	11	SB	482	−	B	1.54	−	−	Y	Y	6−14	Y	N	N	N
20	G	18	SB	480	−	B	1.54	−	−	Y	Y	6−14	Y	N	N	N
21	G	6	SB	602	−	B	1.54	−	−	Y	Y	6−14	Y	N	N	N
22	M	4	SB	508	−	B	1.54	−	−	Y	Y	14−20	Y	N	N	N
23	G	11	SB	482	−	B	1.54	−	−	Y	Y	14−20	Y	N	N	N
24	G	18	SB	480	−	B	1.54	−	−	Y	Y	14−20	Y	N	N	N
25	G	6	SB	602	−	B	1.54	−	−	Y	Y	14−20	Y	N	N	N

**Horse 1–13:** 13 endurance horses: G (*n* = 7), M (*n* = 6). Arab include part Arabian horses, QH, quarterhorse; TB, thoroughbred, endurance exercise; **Horse 14–25**: 12 untrained trotters, all Standardbred (SB): G (*n* = 12), fast trot exercise; G, gelding; M, mare. Gr, gray; C, chestnut; B, bay; The riders’ and horses’ performance history includes: age start, indicates age (years) when horse started competing; horse experience, indicates number of years in competition (40 km or more); GI pill, gastrointestinal pill; GPS, global positioning system; HR, heartrate monitor (Polar); BOM (2020); Bureau of Meteorology, local stations closest to location of exercise at varying km distance from the actual event in total 4 endurance locations and 1 trotter racetrack, distance ranged from 5.3 to 53 km; BOM stations were: Roseworthy 27.9 km distance from ride at Sandy Creek; Rosedale station 5.3 km from ride at Tarlee; Meningie station 53 km from ride at Coorong; Nuriootpa station 28.8 km from ride at Sedan; Roseworthy station at 2.2 km from trotter racetrack; N, no; Y, yes; NK, not known; ^∧^indicates HR only; ^∧∧^indicates second 40 km only; blood gas analysis included pH, pCO_2_, pO_2_, HCO_3_^–^, base excess and lactate levels; **indicates biochemistry by VETSCAN (utilizes *dry* and liquid reagents); TS, total solids; TP, total protein; AST, Aspartate amino transferase; CK, creatinine kinase.

All 13 endurance horses (Horses 1–13, Table 1) were sourced on a voluntary basis through the South Australian Endurance Riders Association (SAERA) at a maximum of four horses per event. All horse owners signed a written consent form before enrolment. The horses remained under the care of their owners for the duration of the events and owners could withdraw their horse at any time during the study. Before competition, all horses were subjected to a health inspection conducted by endurance veterinarians according to AERA riding rules (AERA, 2020).

The second study group consisting of 12 unconditioned trotter horses (Standardbreds; Horses 14–26, Table 1) was randomly selected from a university-based teaching herd at a maximum of four horses per event. Trotters were kept in large paddocks without additional exercise or training. Before enrolment, trotters were subjected to a standard clinical health check. The present study was approved by the University of Adelaide Animal Ethics Committee (project number S-2011-224), which conformed to the Australian Code of Practice for the care and use of animals for scientific purposes, Canberra, Australia, 2004.

### Study Design

The study design is illustrated in Figure 1. Endurance horses (Table 1) were competing at distances of either 40 km (*n* = 2) or 80 km (*n* = 11) and two of those horses continued to cover 100 km. Data collection for endurance horses was performed during four endurance events during the cooler South Australian winter months (May, June, and July) and event locations varied from −34°28′5.70′′ S to −34°32′3.08′′ S. The horses exercised under solar radiation through farming land of varying terrain with altitudes ranging from 4 to 462 m.

Following each exercise loop of 40 km, endurance horses were required to rest for 60 min according to AERA riding rules (AERA, 2020) and were subsequently inspected by independent endurance veterinarians before competition could be continued. Following every exercise loop, blood samples were collected. Subsequently, horses were cooled by pouring buckets of water over their bodies and then scraping it off. Horses were allowed to drink water and eat hay *ad libitum*.

The trotters were harnessed to a jogger and exercised under full sunlight over a 770 m sand training track in South Australia (−34°32′3.08′′ S) during Australian winter months (June, July). The racetrack was harrowed and watered on a regular basis to keep the track conditions as constant as possible. Trotters were subjected to three consecutive phases of exercise: slow warm-up phase (770 m at 25 km h^–1^, not included in data analysis); moderate warm-up phase: 1,540 m at a speed of 35 km h^–1^ (9.7 m s^–1^); and the more intense fast trotter exercise: 1,540 m at a speed of 42–50 km h^–1^ (11.7–13.9 m s^–1^; Figure 1). During the last phase, trotters were encouraged to exercise at their individual maximum speed. Post-exercise, blood samples were collected followed by cooling with a water hose for approximately 10 min and horses were allowed to drink water *ad libitum*. The T_*c*_ recovery period varied from 32 to 83 min depending on the duration of the individual T_*c*_ recovery.

### Continuous Monitoring of GI Temperature Over Time

All horses were equipped with monitoring equipment (Table 1). The GI T_*c*_ representing the core temperature was recorded continuously during exercise and recovery using the ingestible telemetric GI pill (Jonah pill^®^; Phillips, United States. The night before undertaking exercise, GI pills were successfully administered to eight endurance horses and 12 trotters using nasogastric intubation as previously described (Verdegaal et al., 2017). The timing of the GI pill administration was random, unrelated to the horses’ feeding regime.

**FIGURE 2:**
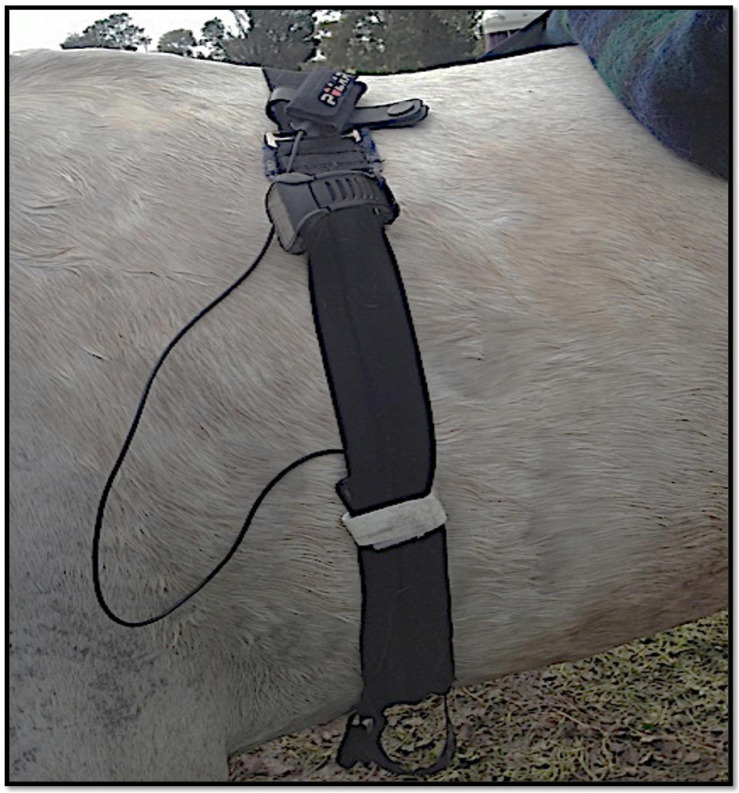
Monitoring equipment: A modified Equivital belt for use on horses with the external receiver device (Sensor Electronics Module) located inside of the belt at of the ventral part of the thorax to monitor the Tc by GI pill, while the Garmin GPS watch and the Polar heartrate electrodes were attached at the upper part of the belt.

Temperature was recorded by an external receiver, Equivital^®^ Sensor Electronics Module (SEM, EQ02 Equivital data Logger^®^, Hidalgo, United Kingdom. The receiver was positioned in a pocket of the Equivital Sensor Belt^®^. This system was modified to accommodate a sturdy strap fitted closely around each horse’s saddle girth (Figure 2). Equivital T_*c*_ data were recorded every 15 seconds and were uploaded from the SEM to the Equivital Software Manager^®^ for data processing.

### Monitoring of Distance, Speed, Inclination, and Heartrate Over Time

Traveled distance, speed, and inclination of the course were recorded every second using a GPS monitor (Garmin Forerunner 910XT GPS watch^®^; Garmin Ltd., Schaffhausen, Switzerland) attached to the gullet of the saddle. The HR was continuously monitored telemetrically by the Garmin watch using Polar electrodes (Polar Electro^®^, Kempele, Finland) and recorded every second (Parker et al., 2009). The GPS and HR data were uploaded from the Garmin watch to the Garmin Connect and Garmin Training Centre^1^.

### Ambient Environment

On each data collection day, ambient temperature (T_*a*_) was recorded continuously in a shaded area of the rest area (post-exercise) using a data logger device (Onset HOBO Pro V2 logger temp U23-00^®^, Onset Computer Corporation, Bourne, ME, United States). In addition, T_*a*_ data were obtained from the Bureau of Meteorology as presented in Table 1.

### Blood Sampling and Analysis

Blood samples from endurance horses were analyzed using portable equipment in the field and data are presented in Table 1. Venous blood was collected (by SJ, E-LV, or LF) from the left jugular vein by needle and divided in two separate syringes within 3 min after exercise. The first blood sample was collected in a 3 ml syringe containing heparin and closed with an airtight seal for venous blood gas analysis using a portable blood gas analyzer (EPOC, Epocal^®^; Ottawa, Canada; Averay et al., 2014; Kirsch et al., 2019). The second venous blood sample was collected in a 20 ml syringe and distributed into a lithium heparin blood tube and a plain blood tube and stored on ice before samples were centrifuged at 3,000 *g* for 10 min (Hematocrit centrifuge 200^®^; Hettich Lab, Tuttlinger, Germany). In trotters, venous blood samples were collected from the left jugular vein by a vacutainer technique in lithium heparin tubes (presented in Table 1).

Hematocrit was determined with the microhematocrit method and plasma total solids via a refractometry meter [Refractometer, RHC-200 (ATC)^®^]. Plasma lactate concentration was analyzed with a hand-held lactate analyzer (Accutrend Plus^®^, Roche, United States). Blood pH recording in trotters was measured using approximately 2 ml of blood and a pH meter (pH Cube^®^; TPS Pty Ltd, Brendale, Australia) with a pH calibration prior to every measurement by a voltage plot against the specified pH value of the buffer solution followed by introduction of the probe into the blood for approximately 30 s to read the pH. The biochemistry profiles were analyzed using a blood gas analyzer EPOC^®^ [*n* = 9; sodium (Na^+^), potassium (K^+^), calcium (Ca^2+^), glucose] or a dry biochemistry analyzer (Vetscan VS2^®^; Chemistry Analyzer, Abaxis, United States; *n* = 4; Na^+^, K^+^, Ca^2+^, glucose, creatinine, urea, aspartate amino transferase, creatinine kinase). Hematology tests were performed in four horses (VETSCAN HM5 Hematology Analyzer^®^, Abaxis, United States). An overview of the blood analysis performed in individual horses is presented in Table 1.

### Data Processing

Each exercise period of 40 km (endurance) or 1,540 m (trotter) will be referred to throughout the study as the T_*c*_ exercise period. Each recovery period (post-exercise) following 40 km (endurance) or 1,540 m (trotters) will be referred to as T_*c*_ recovery period. Recordings performed during each T_*c*_ period included GI temperature (T_*c*_), speed, HR and post-exercise blood values. Hyperthermia was defined as T_*c*_ above 39°C based upon the results of our previous study (Verdegaal et al., 2017) in which the mean maximum T_*c*_ was 38.9°C (95% CI, 38.8–38.9°C) at 6 min post-exercise. A T_*c*_ time profile curve of the individual horse was constructed from the endurance exercise, warm-up exercise (trotters) and (fast) trotter exercise. This included assessment of the value and time point of the maximum T_*c*_ (max T_*c*_) during the T_*c*_ exercise or recovery period (40 km, total 80 km, or 1,540 m), the HR and speed at max T_*c*_, recovery time (min) from max T_*c*_ to a T_*c*_ of 38.5, 38.3, and 38.0°C, respectively, and recovery to the baseline T_*c*_ and number of horses with hyperthermia. In addition, the delta T_*c*_ (°C change post-exercise) and the recovery time from max T_*c*_ to a HR of 60 bpm were calculated as per Australian regulations (AERA, 2020).

To quantify the T_*c*_ response, the net area under the T_*c*_ curve (net AUC) was calculated for T_*c*_ and summated for the total T_*c*_ exercise period and the total T_*c*_ recovery period (Horswill et al., 2008; Datta et al., 2021). The net AUC (baseline set at rest T_*c*_) was calculated using the trapezoidal method of T_*c*_ over time (min) expressed as °C × min. In this study, the net AUC is summated to present the cumulative T_*c*_ – time distribution (Datta et al., 2021). The net AUC provides an estimate of the dynamic thermal response to thermal load. The thermal load takes into account exercise intensity, T_*a*_ including solar radiation, and environmental evaporative power during exercise and recovery (IUPS Thermal Commission, 2001). Further processing of the recorded data included calculation of the metabolic heat production (H, kJ min^–1^; IUPS Thermal Commission, 2001) and the oxygen cost of transport (COT—the oxygen consumption necessary to travel 1 m) for each endurance horse (Schroter and Marlin, 2002). Calculation of both H and COT provides a multi-perspective view on thermoregulatory challenges represented by different types of exercise. The following formula was used:

$$\text{H}=\dot{\text{V}}{\text{O}}_{2}\times k\times \mathrm{duration}\mathrm{of}\mathrm{exercise}$$

— in which V̇O_2_ represents the oxygen consumption rate expressed in liters per min, *k* stands for heat liberated per liter of oxygen consumed (5 kcal; 21 kJ; Hodgson et al., 1993; McCutcheon and Geor, 2008) and duration of exercise is expressed in min. The rate at which oxygen is consumed in an exercising horse provides a direct indication of its metabolic rate and subsequent heat production (Hodgson et al., 1993). In the current study, V̇O_2_ was estimated by calculating the COT, speed and body mass as follows:

$$\dot{\text{V}}{\text{O}}_{2}=\text{COT}\times \mathrm{speed}\times \mathrm{body}\mathrm{mass}$$

— in which COT is the oxygen cost of transport in ml oxygen per kg body mass per m (ml O_2_ kg^–1^ m^–1^), speed is expressed in m min^–1^ and body mass in kg.

The COT is calculated based on an incline, decline, or flat terrain using the following equation (Schroter and Marlin, 2002):

Uphill(incline)andflatterrain:

COT= 0.123+ 1.561(gradient)

Downhillterrain(decline):

COT= 0.123+ 1.591(gradient)+

$$9.762{\left(\text{gradient}\right)}^{2}+14.0{\left(\text{gradient}\right)}^{3}.$$

The gradient of the terrain was recorded as meters incline or decline indicated by the GPS device. The overall H per 40 km T_*c*_ exercise period was calculated by cumulating the H and presented as cumulated H.

### Statistical Analysis

All data are presented as mean ± SD (range). Comparison and correlation analyses were performed using IBM SPSS Statistics 26.0 software or using GraphPad Prism version 9.1.2 for MacOS, GraphPad Software, San Diego, CA, United States^2^. The net AUC (°C × min) and max T_*c*_ (°C) between the first and second 40 km exercise loops were compared using a paired *t*-test and a general linear model ANOVA approach which included horse ID as a random variable. The mean max T_*c*_ was than compared between endurance and trotter exercise using an independent samples *t*-test. The correlation between time to HR ≤ 60 bpm (dependent) and the T_*c*_ (°C) at end of exercise (independent) was assessed by a scatterplot and analyzed using a regression model. Blood values were compared using a one-way ANOVA *post hoc* Tukey’s test. Statistical significance was set at α < 0.05.

## Results

All horses completed their exercise trial without any adverse events. No horses were withdrawn by owners. The owner of horse 13 erroneously removed the GPS equipment during the first 40 km. The GI pills were administered successfully to all horses resulting in complete T_*c*_ recordings. Unfortunately, an exceptional event of a malfunctioning batch of GI pills prevented activation of the GI pill in 5 of the 13 endurance horses. The GPS data were not recorded (only the HR) in 1 endurance horse (horse 7) and in 1 trotter (horse 19) while HR was not recorded in horse 21 (Table 1). Unphysiological and unrealistic values (artifacts) for T_*c*_, GPS and HR (below 10 bpm and above 220 bpm) were removed prior to analysis. The endurance horse group encompassed five mares and seven geldings of different breeds (although mainly Arabians) with an age of 9.5 ± 2.8 years and body mass of 479 ± 68 kg (Table 1). The mean age of starting endurance competitions was 6.1 ± 2.1 years with a mean of 2.5 ± 1.9 years of experience in endurance ride competitions. The T_*c*_ was recorded in eight horses over a total of 80 km T_*c*_ exercise separated in loops of 40 km and associated T_*c*_ recovery periods of 60 min. The Equivital belt became dislodged in horse 1 during the first loop causing data loss at the end of a 40 km loop. As a result, the belt underwent additional modification for the subsequent recordings by fitting sturdy straps sandwiched into the belt to stabilize the girth position. The T_*c*_ was not recorded during recovery following the first loop in horses 1 and 2 due to premature removal of the belt by the owners. The group of 12 trotter horses included 1 mare and 11 geldings with an age of 9.8 ± 5.3 years and body mass of 537 ± 60 kg (Table 1).

### Ambient Environment

On all occasions, the T_*a*_ was relatively cool: mean minimum 6.7 ± 0.4°C, mean maximum 18.4 ± 2.9°C (Table 1). More specifically, the T_*a*_ on the four separate days of endurance exercise showed a minimum T_*a*_ of 13.4, 6.3, 2.8, 6.6°C, and maximum T_*a*_ of 26.3, 19.0, 22.0, 18.8°C, respectively (HOBO data). The T_*a*_ recorded by Bureau of Meteorology stations at varying distances from the event location are presented in Table 1.

### Individual GI Temperature, Heartrate and Speed Data During Endurance and Trotter Exercise Over Time

The individual T_*c*_ parameters and descriptive analysis of T_*c*_ are presented in Supplementary Tables 1, 3. The descriptive analysis of speed, HR, H, and COT (endurance only) and duration of exercise of individual horses is presented in Supplementary Table 2.

### Overall T_*c*_ Profiles During Endurance Exercise

**FIGURE 3:**
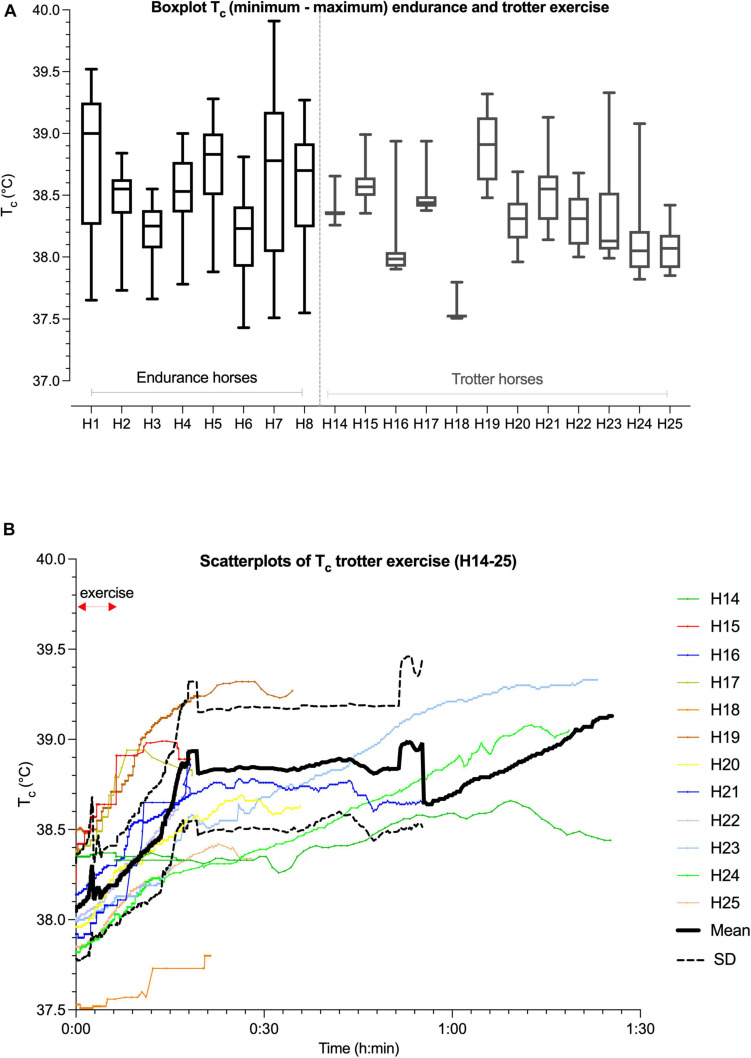
Box and whisker plots demonstrating median (solid line), interquartile range (identified by rectangular area), and minimum to maximum (max) ranges of data (whiskers or error bars) of boxplots of T_*c*_ (°C) in horses (H; horse number) in endurance (H1–H8) and trotter (H14–H25) exercise **(A)**. Scatterplots with T_*c*_ (°C; left *y*-axis) over time [duration of exercise in hours (h) and minutes (min)] (*x*-axis) in trotter horses (H14–25) and its mean (thick black line) ± SD (dotted black line, calculated when *n* > 3 horses) during high intensity trotter exercise over time **(B)**.

**FIGURE 4:**
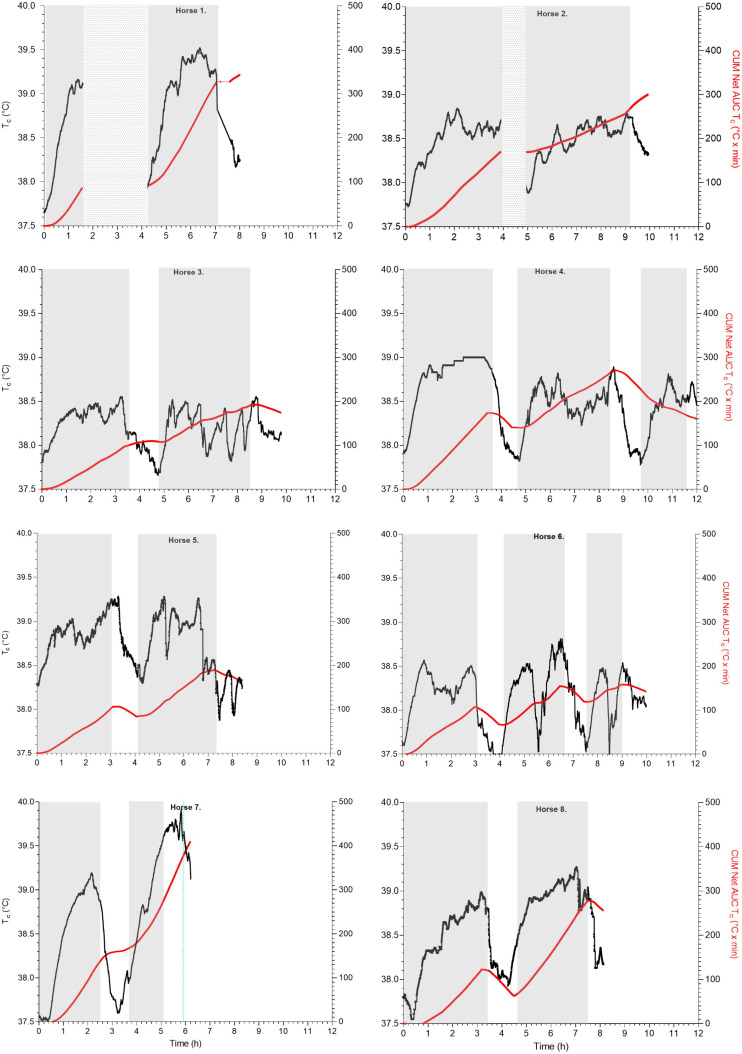
Scatterplots of T_*c*_ (°C; left *y*-axis, black line) and cumulated net AUC (T_*c*_°C × min; right *y*-axis, red line) over time (hours, h) during T_*c*_ exercise periods (identified as gray blocks) and recovery in endurance horses H1–8; H1 data loss at end of leg 1 and H1, H2 without T_*c*_ recording during the first recovery (identified as spotted blocks); H4, H6 continued to 100 km total.

The overall mean speed of endurance horses was 14.0 ± 1.4 km h^–1^ over the first 40 km (*n* = 11) and 14.2 ± 2.1 km h^–1^ over the second 40 km (*n* = 11) with a mean HR of 114 ± 13 bpm (Supplementary Tables 1, 2). There was no significant correlation between end T_*c*_ of the first 40 km endurance exercise loop and recovery time to HR ≤ 60 bpm and (*p* = 0.646) but the end T_*c*_ at the second 40 km showed a significant correlation with HR recovery (*p* = 0.045). The cumulated H of the total 80 km was 120,000 ± 18,000 kJ. The T_*c*_ profiles of endurance horses during exercise and recovery are presented in Table 2, Figures 3A, 4, and Supplementary Tables 1, 2.

**TABLE 2:** Overall variables during exercise and recovery of endurance and trotter exercise.

Type exercise	Endurance (*n* = 16 40 km)	Trotter 2^nd^ warm-up (*n* = 12 1,540 m)	Trotter (*n* = 12 1,540 m)
Duration (min) exercise	198 ± 63 (83–247; 40 km, *n* = 25); T_*c*_ only: 191 ± 43 (*n* = 16)	3.7 ± 0.4	2.9 ± 1.4. (1.5–6.1)
Duration (min) recovery	60	4.9 ± 1.4 (2.6–7)	40.2 ± 30.2 (11.5–83)
T_*c*_ (°C)	38.5 ± 0.3 (38.2–39.0)	37.9 ± 0.3 (37.5–38.3)	38.1 ± 0.3 (37.5–38.5)
Max T_*c*_ (°C; during exercise or recovery)	39.0 ± 0.4 (38.5–39.9**)	38.0 ± 0.3**	38.8 ± 0.5 (37.6–39.3)**
Time to max T_*c*_ (min)	143 ± 60 (54 – 245, *n* = 15)	NA**	34.3 ± 28.4 (6.5–79)**
Exercise distance (km) to max T_*c*_	29.5 ± 11.6 (11–40, *n* = 15)	NA	NA
Max T_*c*_ ≥ 39°C	8/16	0	4/12
Max T_*c*_ ≥ 38.5°C	All	0	5/12
Speed at max T_*c*_ (km h^–1^)	16.1 ± 4.3 (4.7–21)	22.5 ± 8.3	337.7 ± 10.0 (18.6–51.9)
HR at max T_*c*_ (bpm)	105 ± 29 (63–152)	135.1 ± 39.0	145 ± 36 (80–204)
Return T_*c*_ to 38.5°C	15/16	6/12	1/12 (and 1 T_*c*_ never above 38)
Time max T_*c*_ to 38.5°C (min)	21.2 ± 22.9 (0–80)	NA	NA
Return T_*c*_ to 38.3°C	13/16	2/12	1/12 (and 1 T_*c*_ never above 38)
Time max T_*c*_ to 38.3°C (min)	31 ± 24.6 (0 ->77; *n* = 13/16)	NA	NA
Return T_*c*_ to 38.0°C	10/16	8/12	8/12 (1 T_*c*_ never above 38)
Time max T_*c*_ to 38.0°C (min)	39.6 ± 14 (*n* = 8/16)	0 (6//12; 2 T_*c*_ never above 38)	NA
Return to base T_*c*_ (Y/N)	4/16	8/12	1//12 (and 1 T_*c*_ never above 38)
Time max T_*c*_ to baseline (min)	52 ± 37 (*n* = 4/16)	0 (4/12); (1 T_*c*_ never above baseline)	NA
Net AUC T_*c*_ exercise (endurance: 2^nd^ 40 km from base T_*c*_)	7,116 ± 1,997 (7,203 ± 3,125)	7.9 ± 11	4.8 ± 8.5
Net AUC T_*c*_ exercise 1^st^ 40 km	77,421 ± 1,940	NA	NA
Net AUC T_*c*_ exercise 2^nd^ 40 km	6,811 ± 2,138	NA	NA
Net AUC T_*c*_ recovery	−1,516 ± 1,326 (*n* = 15)	5.4 ± 6.8	1,821.3 ± 4,434.0 (−14.3–15,769)
Net AUC T_*c*_ recovery 1^st^ 40 km	−1,979 ± 1,555 (*n* = 7)	NA	NA
Net AUC T_*c*_ recovery 2^nd^ 40 km	−1,169 ± 1,102 (*n* = 8)	NA	NA
Delta T_*c*_ first 10 min recovery (°C)	−0.24 ± 0.34 (−0.24–0.15, *n* = 15)	NA	+ 0.36 ± 0.16 (0–0.58; *n* = 11)
Net AUC min^–1^ (°C min^–1^) exercise	37.3 ± 9.9	2 ± 2.7	1.9 ± 3.7
Delta T_*c*_ end exercise to end recovery	−0.71 ± 0.4 (−1.16–0.23)	0.1 ± 0.1 (0–0.2)	0.6 ± 0.4 (0–1.3)
Delta T_*c*_ end exercise to end recovery min^−1^	−0.01 ± 0.01 (−0.02–0.02)	0.02	0.04 ± 0.02 (0–0.06)
Time to HR ≤ 60 bpm (min)	6.3 ± 6.5 (0–24)	2.3 ± 1.2 (0.5–4)	23.1 ± 20.9 (5–51) 4/12
T_*c*_ > 39°C at end recovery	1/16 (39.13 after 90 min)	NA	4/12
Net AUC min^–1^ (°C min^–1^) recovery	−23.7 ± 20.4 (−55–13.8)	2.9 ± 3.3	54.5 ± 143.6
Speed (km h^–1^)	14.1 ± 1.7 (10.5 −17.4; *n* = 22, without horse 7)	34.1 ± 3.5 (30.4–38.0; *n* = 7)	38.2 ± 0.3 (38.0–38.8; *n* = 7)
Max speed (km h^–1^)	23.4 ± 2.8 (19.2–30; *n* = 22)	34.1 ± 7.3 (25.0–42.3; *n* = 7)	49.7 ± 3.9 (42–52.7; *n* = 7)
HR (bpm)	114 ± 13 (89 −143; *n* = 24)	130 ± 32 (75–177; *n* = 8)	151 ± 17 (130–169; *n* = 8)
H (kJ) total 80 km exercise^#^	71,000 ± 12,000 (52,000–94,000; *n* = 22)	–	–
COT (ml O_2_ kg^–1^ m^–1^)^#^	0.13 ± 0.01 (0.11–0.15; *n* = 22)	–	–
V̇O_2_ (LO_2_ min^–1^) ^#^	15.0 ± 2.3 (11.2–20.5; *n* = 22)	–	–

Data are presented as overall mean ± SD. T_*c*_, GI pill temperature; n, number identified only if different to 16 or 12 exercise periods; max T_*c*_, maximum T_*c*_; AUC, area under the curve; ^#^indicates calculated; HR, heartrate; H, metabolic heat production; COT, cost of transfer; V̇O_2_ L min^–1^, oxygen consumption in liters per min; *indicates during exercise; **indicates during recovery; NA, not applicable; –indicates not available.

The mean max T_*c*_, absolute max T_*c*_, and net AUC of the first 40 km exercise were not significantly different from the second 40 km exercise [*F*_1_,_7_ = 2.436, *p* = 0.163 (for max T_*c*_), *p* = 0.66 (for max absolute T_*c*_), and *p* = 0.56 (for net AUC), respectively; Figures 3A, 6]. Mean duration of all 40 km T_*c*_ endurance exercise was 191 ± 43 min. The mean max T_*c*_ during exercise was 39.0 ± 0.4°C and time to max T_*c*_ was 143 ± 60 min at a mean distance of 29.5 ± 11.6 km. Hyperthermia was recorded in 50% of the T_*c*_ exercise periods. The total dynamic thermal response (net AUC, T_*c*_ °C × min) was 106.4 ± 44.3°C × min per 40 km and was approximately 0.5°C per min (Figure 6).

### Overall T_*c*_ Profiles During Endurance Recovery

Post-exercise, only 25% of all T_*c*_ recovery periods returned to their base T_*c*_ while 50% decreased to or lower than 38°C within 39.6 ± 14 min. The time that T_*c*_ returned to 38.5°C varied between 0 and 57 min (14/15 T_*c*_ recovery periods). Horse 7 showed a different exercise T_*c*_ time profile with an end of exercise T_*c*_ of 39.4°C while the max T_*c*_ (39.9°C) occurred at 57 min post-exercise (Figure 4). The dynamic thermal response during T_*c*_ recovery period demonstrated a heat loss by a negative value (−18.1 ± 25.9°C × min) and a large variation amongst individuals (range 23.7–59.7°C × min) with a net AUC min^–1^ −0.3 ± 0.4°C.

### Overall T_*c*_ Profiles During Moderate Warm-Up Trotter Exercise and Recovery

**FIGURE 5:**
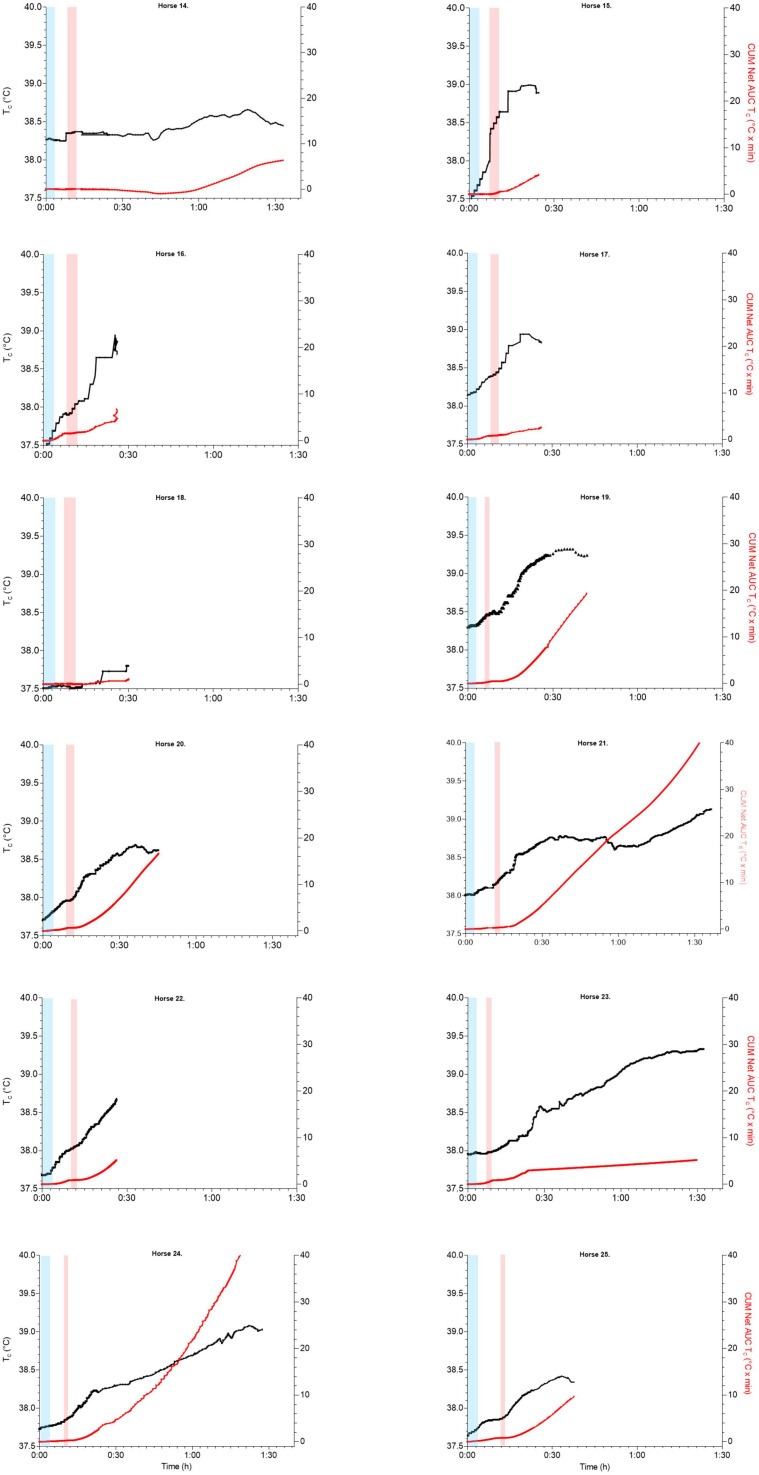
Scatterplots of T_*c*_ (°C; left *y*-axis, black line) and cumulated net AUC (T_*c*_, °C × min; right *y*-axis, red line) over time (hours, h) during exercise and recovery in trotter horses H14–25, warm-up exercise (moderate intensity, identified as blue blocks), and trotter exercise (high intensity, identified as light-red blocks).

The T_*c*_ profiles of trotter horses during exercise and recovery are presented in Table 2, Figures 3, 5, Supplementary Tables 1, 2. The mean speed of the trotters during 1,540 m distance trotter exercise was 27.3 ± 5.3 km h^–1^ with a mean HR of 140 ± 24 bpm (Supplementary Table 2). The T_*c*_ profile of trotter moderate exercise revealed a minor T_*c*_ increase and a minor dynamic thermal response during exercise and during recovery.

### Overall T_*c*_ Profiles During Trotter Exercise

The mean speed of the trotters during 1,540 m distance trotter exercise was 40.0 ± 2.9 km h^–1^ with a mean HR of 147 ± 17 bpm (Supplementary Table 2).

### Overall T_*c*_ Profiles During Trotter Recovery

**FIGURE 6:**
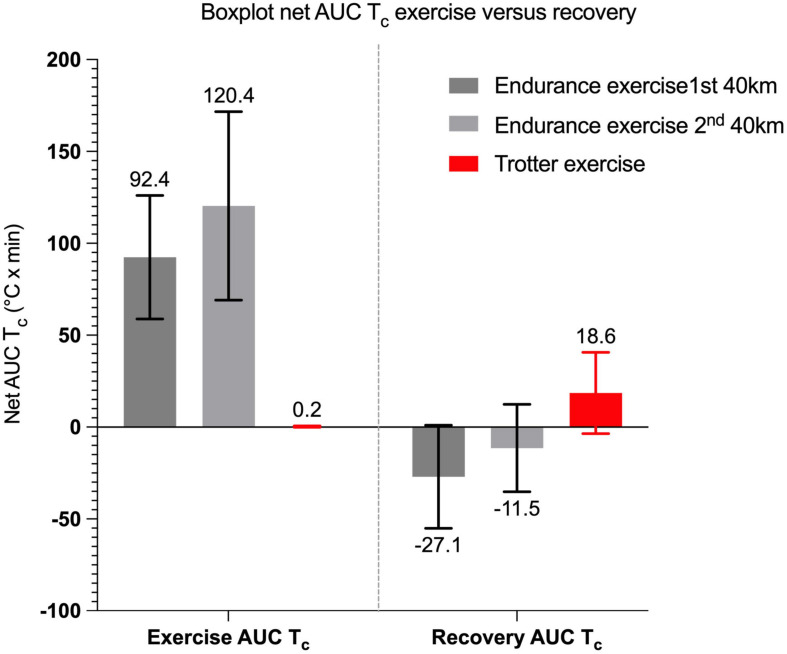
Boxplot of the dynamic thermal response presented by net AUC T_*c*_ (°C × min) during T_*c*_ exercise period of endurance and trotter exercises (presented with mean net AUC on top bar). The 1^st^ 40 km of endurance exercise was not significantly different to the 2^nd^ 40 km. Net AUC T_*c*_ of exercise and recovery from full speed trotter exercise over 1,540 m was minor during exercise while T_*c*_ increased post-exercise as represented by positive net AUC T_*c*_.

Post-exercise, HR recovered to 60 bpm in 5 of the 12 trotters and was coupled with a T_*c*_ lower than 39.0°C in all those trotters. The T_*c*_ profile of trotters reached their mean max T_*c*_ of 38.8 ± 0.5°C during recovery within a mean time of 40.2 ± 30.2 min. The overall increase in mean T_*c*_ (delta) varied (0.5 ± 0.5°C). Hyperthermia occurred in more than 40% of the trotters. Indeed, four trotters still had a T_*c*_ higher than 39°C at the end of recovery, mean time of 68.6 ± 24.6 min duration. Only one T_*c*_ returned to 38°C baseline during recovery. The T_*c*_ profiles of trotter horses during exercise and recovery are presented in Table 2, Figures 3, 5, and Supplementary Table 1. The dynamic thermal response was low during trotter exercise with a mean of 0.2 ± 0.4°C. However, thermoregulation in trotters occurred during recovery with a mean dynamic thermal response of 18.6 ± 22.1°C × min with a wide range (Figure 6) and 0.5 ± 0.3°C net AUC min^–1^ duration of recovery (Supplementary Table 3).

### Comparison of the T_*c*_ Profiles During Endurance and Trotter Exercise

The mean overall values of all data during the two different types of exercise are presented in Supplementary Table 1. The max T_*c*_ in endurance exercise did not significantly differ from max T_*c*_ in trotter exercise (*p* = 0.19). In addition, the max T_*c*_ and absolute max T_*c*_ of the first 40 km and second 40 km endurance exercise loops did not significantly differ from trotter exercise when analyzed separately using an independent sample *t*-test (*p* = 0.49 and *p* = 0.26, respectively).

After 60 min recovery, hyperthermia persisted in only one endurance horse performing endurance exercise. This is in contrast to trotter exercise during which hyperthermia occurred during recovery in 5 of the 12 trotters.

### Blood Analysis Data in Endurance and Trotter Exercise

**FIGURE 7:**
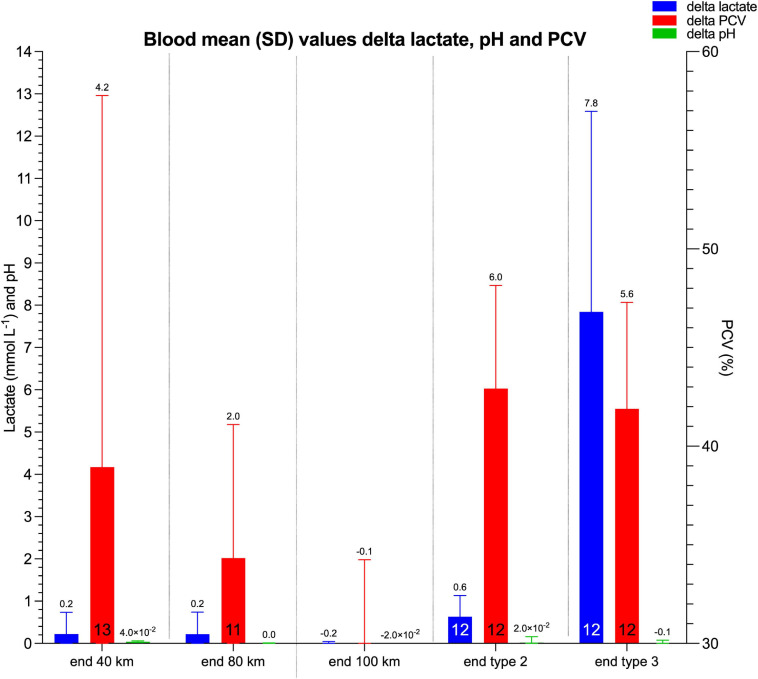
Bar graph of blood parameters: lactate and pH on the *y*-axis presented as mmol L^–1^ and the number, respectively, and the PCV (Hct) on the right y-axis presented as %. The type of exercise is presented on the *x*-axis as pre-, and post-ride in endurance and trotter exercises. The mean value is presented on top of the bar while sample size at the bottom in column.

Blood results and the delta of lactate, pH, and Hct values of all horses (endurance and trotter exercise) are presented in Figure 7, Supplementary Table 3, and Supplementary Figure 1. An ANOVA revealed a significant difference in post-exercise delta lactate value between endurance (1.2 ± 0.6 mmol L^–1^) and trotter (9.4 ± 4.0 mmol L^–1^) as well as between warm-up trotter (1.6 ± 1.0 mmol L^–1^) and fast trotter exercise with a higher lactate value in the last exercise [*F*_(__2_,_38__)_ = 37.63, *p* < 0.0001]. No significant difference was identified between first and second 40 km endurance exercise (*p* = 0.984), however, comparing both first and second 40 km endurance horses to trotter horses separately confirmed a highly significant difference (*p* < 0.001). In addition, mean delta lactate in the 40 km endurance exercise was significantly different to trotter exercise using an independent samples *t*-test (*p* < 0.001).

The delta Hct and delta pH values did not reveal significant differences between endurance and trotter exercise (*p* = 0.60 and *p* = 0.26, respectively).

## Discussion

This is the first study to be conducted using a telemetric GI pill in field competitions to compare the equine thermoregulatory response to different types of exercise under normal weather conditions during the cooler South Australian months. For this purpose, a large dataset of T_*c*_ recordings and other physiological responses was analyzed and compared between two types of exercise that differed greatly in duration and intensity (endurance compared with trotter exercise). As a result, an integrative real time view was obtained on how the equine body copes with heat load production during different types of exercise in the field. The GI pill proved to be a reliable tool to continuously monitor individual thermoregulatory well-being in sport horses.

There was an important difference in the time profile of the thermoregulatory response between endurance and trotter horses. Endurance horses reached their mean max T_*c*_ during exercise toward the end of each leg of 40 km (after ± 30 km) and almost all returned to a T_*c*_ lower than 38.5°C during 60 min into recovery. Trotter horses, however, always reached their mean max T_*c*_ post-exercise at least 30 min into recovery. Moreover, in a third of the trotters, T_*c*_ was still higher than 39°C and HR was higher than 60 bpm at the end of recovery. When HR is at 60 bpm, horses may still have a high T_*c*_ (38.8 ± 0.4°C, range 38.0–39.7°C). Continuous monitoring of T_*c*_ by a telemetric temperature-sensitive pill that passes through the GI tract has been evaluated in horses previously during rest and 158 km transport using the CorTemp^®^ system (Green et al., 2005, 2008). While the Equivital system was validated in our previous study similarly during rest periods (Verdegaal et al., 2017), we applied this monitoring method in equine athletes specifically for the first time during field exercise.

The current study showed that the dynamic thermal response calculated by the net AUC proved to be a more useful presentation of the metabolic response rate than the calculated H (based on calculated values). Despite the individual variation of the max T_*c*_ in endurance and trotter horses, it is of interest that the overall max T_*c*_ in endurance horses (39.0 ± 0.4°C; range 38.5–39.9°C) was not significantly different to the max T_*c*_ in trotters (38.8 ± 0.5°C; range 37.6–39.3°C). Another important finding was that persisting hyperthermia was recorded in four trotters after a mean of 69 min post-exercise while only one endurance horse recorded 39.9°C after 60 min of recovery.

Most importantly, the current study has demonstrated that horses show important individual differences in their thermoregulatory response to different types of exercise. This result was obtained due to the continuous monitoring of the T_*c*_ instead of performing serial measurements of T_*re*_. For example, there was a large variation (range 11–40 km) between endurance horses with respect to the distance at which max T_*c*_ was reached. It is noted that while the moment-to-moment location of the GI pill is unknown, based on a previously performed study we can state that T_*c*_ changes are mainly attributed to exercise and less likely to the movement of the GI pill along the GI tract (Verdegaal et al., 2017). Both the individual variation in max T_*c*_ and dynamic thermal response underline that individual continuous T_*c*_ monitoring is essential for reliable oversight. This reliability is especially important since many horse-specific factors contribute to the thermoregulatory response, such as the genetic make-up of a horse, its temperament and its basic performance capacity level. However, for endurance horses, their performance capacity levels should have been quite similar for all studied horses, keeping in mind their similar competition experience levels for the 80 km distance (Table 1).

### Continuous Individual Monitoring Studies of the Thermoregulatory Response to Date

Based on recent equine and human studies, it is important to review different tools that allow for continuous reliable monitoring of the thermoregulatory response to exercise in the field with a minimal data loss. In that respect, the GI pill has proven to be a practical, accurate, and precise tool to monitor thermal response during field exercise both in human and equine athletes (Edwards and Clark, 2006; Verdegaal et al., 2017; Olcina et al., 2019).

A recent alternative method is the use of intra-muscular microchips embedded in different locations to continuously record muscle temperature (Kang et al., 2020). Although the study was performed under laboratory conditions, this technique could eventually be employed in the field. There was a positive correlation between muscle temperature and central venous temperature (CVT) only when combining all exercise phases including recovery. Interestingly during the recovery phase, the muscle temperature continued increasing and lagged behind the CVT. This non-alignment is in accordance with previous studies reporting on the lack of direct correlation between muscle temperature and core temperature (Byrd et al., 1989; McCutcheon et al., 1992; Hodgson et al., 1994). Therefore, more research is needed to obtain a better understanding of the associations between muscle temperature and core body temperature. One possible disadvantage of the technique is the requirement of invasive surgery to place the microchip.

### Comparison of the T_*c*_ Profiles During Endurance and Trotter Exercise

The results of the current study are important for formulation of future competition and hot weather policies (MOOC endurance, 2021; MOOC heat illness, 2021). The study results show that both endurance and trotter horses reached comparable max T_*c*_, although at different time points. Endurance horses reached their mean max T_*c*_ (39.0 ± 0.3°C, 38.5–39.5°C) during endurance exercise toward the end of each leg of 40 km (after ± 30 km) and almost all T_*c*_ returned to lower than 38.5°C during 60 min into recovery. In contrast, after the completion of trotter exercise, trotter horses reached their max T_*c*_ (38.8 ± 0.5°C, 37.6–39.3°C) at an average of 34 min post-exercise with a mean T_*c*_ increase of 0.6 ± 0.4°C (0–1.3°C). Moreover, in a third of the trotters, T_*c*_ was still higher than 39°C and HR was higher than 60 bpm at the end of recovery. We found that within the 60-min rest time, all endurance horses achieved an overall recovery to 38.5°C in 15 out of 16 T_*c*_ 40 km exercise periods, and 50% of these horses reached lower than 38°C. This supports the mandatory rest periods of 60 min as determined in the AERA and FEI endurance regulations when exercising in a cool environment (FEI, 2018; AERA, 2020). The present study was not able to demonstrate a correlation between the end T_*c*_ first 40 km and recovery time to HR of 60 bpm but the end T_*c*_ second 40 km was related to HR recovery. According to the AERA competition regulations, horses with a HR of 60 bpm at the end of their recovery period would meet all criteria to be labeled as “fit to continue” the competition while interestingly, we found the mean overall 40 km T_*c*_ was still higher than normal (38.8 ± 0.4°C; range 38.0–39.7°C). This means that at the time that the HR recovered to 60 bpm, most horses were still actively coping with increased core body temperature. FEI regulations are stricter than AERA rules regarding the duration of the HR recovery time and require the HR to return below 64 bpm within 15 min post-exercise (FEI, 2021). Our study demonstrated that in the majority of T_*c*_ periods (20 out of 22, T_*c*_ 40 km exercise periods) the HR recovered to below 64 bpm within 15 min but again, the mean T_*c*_ was still 38.7 ± 0.4°C (range 38.0–39.6°C; FEI, 2021). The HR recovery regulations in endurance horses are based on studies demonstrating that not only increased speed and distance but also a longer than 11–13 min cardiac recovery time is associated with higher risk of metabolic elimination after endurance exercise, more specifically with a 70% probability of elimination at the next veterinary check (Nagy et al., 2010; Younes et al., 2015; Bennet and Parkin, 2018). More field research is needed to investigate when heat accumulation in such horses can become problematic during follow-up exercise. The difference in thermoregulatory responses between endurance exercise and racing exercise is well-known and the current study provides exercise-specific core thermal response features which need to be addressed when formulating future equine competition and hot weather policies.

### Hyperthermia Post-exercise

Overall, 50% of the endurance horses developed hyperthermia during the standardized exercise loops, which underlines the importance of an effective T_*c*_ recovery period of sufficient duration. The persistence of hyperthermia in exercising endurance horses is one of the several factors causing metabolic disorders and fatigue due to the decreased supply of blood and fuel to the exercising muscles, brain and GI tract, and increased muscle and brain temperature (McCutcheon et al., 1992; Hodgson et al., 1993; Jones and Carlson, 1995; Geor and McCutcheon, 1998; Gonzalez-Alonso et al., 1999; McConaghy et al., 2002; Gerard et al., 2013; Brownlow et al., 2016; Brownlow and Mizzi, 2021).

While hyperthermia and heat stress are more commonly associated with prolonged exercise, we have demonstrated that horses performing short bouts of strenuous exercise in the field may suffer increasing from hyperthermia during recovery (1/3 of the trotters, Figures 3B, 5 and Supplementary Table 1).

Hyperthermia in racehorses post-exercise can be explained by the greater amount of metabolic heat generated by their larger muscle mass (compared to endurance horses) during their shorter exercise duration but higher intensity exercise (Hodgson et al., 1994; Geor et al., 1995; Jones and Carlson, 1995; Schott et al., 1999). In addition, the high intensity trotter exercise represents a short but acutely pronounced challenge to a multitude of organ systems at the same time such as the respiratory, cardiovascular, musculoskeletal and sympathetic nervous systems, of which the latter may be one of the driving forces behind the hyperthermia (Hetem et al., 2013). The “acuteness” may also explain the hyperthermia that was encountered in more trotter horses compared to endurance horses as well as the more significant interindividual variation seen at the level of the thermoregulatory response in trotter horses. Finally, the current study results underline the importance for formulating guidelines for the management and welfare of all equine sports disciplines. These physiological factors, coupled with environmental factors in the field, can overwhelm the capacity of the heat loss mechanisms of sport horses. It is important to point out that there is no scientific evidence to date suggesting that horses experience heat injury when their T_*c*_ reaches and is sustained at 39°C. However, it should be kept in mind that if horses, and especially trotters, engage in a subsequent exercise bout too soon, their core body temperatures would be starting at an elevated level.

In most trotters (7/12), the first signs toward lowering and normalizing core body temperature became visible after a minimum of 20 min into the recovery. Problems may arise when the hyperthermia in racehorses (with or without clinical signs) is not recognized. The delayed onset of hyperthermia may go unnoticed when trotters are transported shortly after completion of the race. Hyperthermia coupled with transport during hot weather is likely to increase heat loading. The identification of ongoing hyperthermia during recovery is essential information with respect to equine welfare and emphasizes the need for extended continuous monitoring post-exercise. Our findings may explain personal anecdotal evidence expressed by trotter trainers noticing that some horses show discomfort the day after an otherwise uneventful competition day (Courouce et al., 2002; Richard et al., 2010; Bertuglia et al., 2014) due to unrecognized post-racing hyperthermia (McConaghy et al., 2002; Brownlow and Mizzi, 2020, 2021). The current findings also underline that the habit of withholding water prior to racing events is not an ideal practice. Indeed, several studies involving endurance or trotter horses report that dehydration and electrolyte loss are significant predictors for early elimination due to metabolic reasons (Barton et al., 2003; Fielding et al., 2009; Barnes et al., 2010; Munoz et al., 2010; Trigo et al., 2010; Waller and Lindinger, 2010). Likewise, a recent meta-analysis in human athletes demonstrated the importance of fluid ingestion to counteract hyperthermia and to improve performance capacity (Alhadad et al., 2019). In our study, no fluid intake restrictions were applied and blood values showed that the studied horses were not dehydrated.

As in endurance horses, the recovery for trotters to HR 60 bpm was not associated with a recovered T_*c*_. This is important knowledge for the monitoring of trotters during recovery when using HR as guidance. At a HR of 60 bpm point in time, the mean T_*c*_ was still 38.7 ± 0.5°C (range 38.0–39.3°C) while 8/12 trotters still measured a T_*c*_ higher than 38.5°C. A post-exercise peak T_*re*_ was earlier reported in a few other studies (Hodgson et al., 1993; Kohn and Hinchcliff, 1995; Marlin et al., 1996, 1998; Kohn et al., 1999; Foreman et al., 2006; Verdegaal et al., 2017) and supports the conclusion that it is necessary to continue monitoring racehorses during recovery. Based on our findings and coupled with those reported by previous studies, continuous monitoring of racehorses and sport horses for hyperthermia using the GI pill or T_*re*_ during a period of rest for at least 60 min post-exercise is highly recommended.

### The Dynamic Thermal Response (Net AUC T_*c*_)

The continuous T_*c*_ monitoring allowed for the construction of detailed temperature-time profiles and the subsequent application of the AUC approach. The present study measured net AUC T_*c*_ to assess the dynamic thermal response to the metabolic thermal load, a product of the thermoregulatory mechanisms that increase temperature over time (Hodgson et al., 1993; Schroter and Marlin, 1995; Schott et al., 1999; Hodgson, 2014). As expected, our study demonstrated that the mean dynamic thermal response was much higher during endurance exercise compared to trotters due to higher H over time. The core thermal response during recovery in endurance horses was negative due to metabolic heat dissipation. Alternatively in trotters, both heat load and heat loss mechanisms were in place during recovery, represented by a relatively high dynamic thermal response compared to the short duration of the exercise. The thermal response index using area under the fever curve has been applied in several mammal studies comparing the fever response over time (Feng and Lipton, 1987; Murakami and Ono, 1987). Only a few equine research groups report the use of AUC to assess temperature response over time. One equine study used AUC to demonstrate changes in muscle and tendon tissue temperature over time during therapeutic ultrasound treatments at different tissue depths (Montgomery et al., 2013). Studies involving human athletes have used AUC to compare AUC temperature response in two study groups. One study used AUC to quantify the body temperature response over time (60 min cycle exercise at 65% $\dot{\text{V}}$O_2_max) by continuous recording of T_*re*_ and demonstrated that T_*re*_ AUC response was not different between ingesting a carbohydrate drink [47.7 ± 11.6 (°C × min)] and a placebo [50.1 ± 11.1 (°C × min)]—unfortunately recovery data were not reported (Horswill et al., 2008). Another study reviewed comparisons of different cooling methods using AUC (T_*re*_ to time) to assess cooling rates over time and argued that a larger AUC was associated with an increased risk of tissue and organ injury (Casa et al., 2007). A recent human study used AUC to quantify the thermal response during bladder cancer treatment to assess its use as a prognostic parameter and concluded AUC was a simple calculation to predict the medical outcome (Datta et al., 2021). Similarly, we can argue that in our study a high net AUC (high dynamic thermal response) may be associated with a higher risk of thermally based injury due to insufficient thermoregulatory mechanisms. When comparing the values of the calculated H and calculated COT in our study across endurance exercise (according to Schroter and Marlin, 2002), the H calculations were not discriminative for endurance horses with high max T_*c*_. Moreover, these calculations did not consider anaerobic exercise and therefore could not be applied to trotter exercise for comparison. Another concern is that the H equation is formula-based using several calculated values such as V̇O_2_. When comparing this calculated H method to the AUC method which can only be used in association with a continuous monitoring technique, the latter seems a much more accurate approach.

### Blood Values in Response to Exercise

Blood lactate values showed only a mild increase in endurance horses and were not different between the first and second 40 km endurance exercise. These values were similar to an earlier report which identified no difference in blood lactate values between eliminated endurance horses and finishers (Fielding et al., 2009).

## Limitations

Our current study has various limitations that should be considered when assessing our findings. As applies in all field studies, not all conditions could be 100% controlled, such as perfectly equal conditions of competition tracks, T_*a*_, solar radiation, and the fact that the endurance part involved privately owned horses which obviously received different diets and followed different trainings protocols. In retrospect, scraping off water from the horses during cooling down was not the most optimal approach since recent research comparing five cooling methods in racehorses favored continuous reapplication of cold water without subsequently scraping it off (Takahashi et al., 2020). We did not seek to investigate individual external horse-related influencing factors such as diet, supplements, health history, duration of transport prior to competition nor processes such as the circadian rhythms which may have caused a diurnal variation in T_*c*_. Future research is needed to investigate these variables to augment our understanding of heat load production in sport horses in varying ambient conditions.

## Conclusion

Continuous monitoring of T_*c*_ using the GI telemetry pill is a reliable, non-invasive method of assessing the thermoregulatory response in exercising horses. It provides vital individual recording and monitoring of important interindividual differences in T_*c*_ time profiles. Clearly, seeking a universal thermoregulatory response pattern that will apply to all exercising horses will prove futile. Consequently, continuous monitoring allows for applying an AUC approach which is a more robust parameter when compared to calculated H. In our study, endurance horses reached their max T_*c*_ on average when completing over 75% of a 40 km exercise leg; this returned to baseline within the mandatory 60 min recovery time. However, several horses still had T_*c*_ values above 38.5°C when their HR had already returned to 60 bpm which is used in endurance (AERA) as “fit to continue” competing. Trotter horses reached their peak T_*c*_ during their recovery period on average at 34 min after exercise. Here again, several horses had T_*c*_ values above 38.5°C with a HR already returned to 60 bpm. Our results have shown that T_*c*_ monitoring should continue in trotter horses at least until 60 min post-exercise.

Future research involving strenuously exercising horses should focus on the possible impact of intrinsic characteristics such as genetics and training, as well as more challenging environmental conditions. Our findings have implications for improving the overall welfare of sport horses and future management of equine welfare at sport events such as the Olympic Games (Hosokawa et al., 2020; Elliott, 2021). Our current study provides reliable supporting evidence for the need for industry-wide, temperature-monitoring guidelines to prevent EHI in endurance horses and racehorses when exercising and recovering in the field.

## Data Availability Statement

The original contributions presented in the study are included in the article/Supplementary Material, further inquiries can be directed to the corresponding author/s.

## Ethics Statement

The animal study was reviewed and approved by University of Adelaide Animal Ethics Committee (project number S-2011-224) Australia. Written informed consent was obtained from the owners for the participation of their animals in this study.

## Author Contributions

E-LV developed the study design and contributed to data collection, preparation, and creation of the database, descriptive and statistical analysis, interpretation of the data, and writing of the manuscript. CD was involved in study design, preparation, and creation of the database, analysis and interpretation of the data, and writing of the manuscript. GH contributed to drafting and revision of the manuscript. SJ was involved in study design and data collection. TM contributed to reviewing the manuscript and statistical analysis. BB and CV were involved in data file setup and reviewing the manuscript. SF was involved in the initial design and contributed to data collection. LF and SF were involved in the initial design of the study and data collection for the trotter horses. All authors read and approved the final manuscript.

## Conflict of Interest

The authors declare that the research was conducted in the absence of any commercial or financial relationships that could be construed as a potential conflict of interest.

## Publisher’s Note

All claims expressed in this article are solely those of the authors and do not necessarily represent those of their affiliated organizations, or those of the publisher, the editors and the reviewers. Any product that may be evaluated in this article, or claim that may be made by its manufacturer, is not guaranteed or endorsed by the publisher.

## Footnotes

1
https://connect.garmin.com/

2
www.graphpad.com

## Supplementary Material

Supplementary Figure 1 Bar graph comparing delta blood parameters lactate, pH and PCV (Hct) pre-, and post-ride in endurance and trotter exercise.

Supplementary Table 1 T_*c*_ parameters in endurance exercise (per 40 km) and trotter exercise.

Supplementary Table 2 Global Positioning System (GPS), heartrate (HR) values and calculated values related to metabolic heat production (H).

Supplementary Table 3 Blood parameters of endurance horses and trotters (only lactate, PCV, pH) pre- and post-ride.

## Abbreviations

GI: Gastrointestinal
COT: Cost of transfer.
