# Supplementary material for: Continuous Monitoring of the Thermoregulatory Response in Endurance Horses and Trotter Horses During Field Exercise: Baselining for Future Hot Weather Studies
Source: Front Physiol. 2021 Aug 26;12:708737. doi: 10.3389/fphys.2021.708737 (PMC8427666; doi:10.3389/fphys.2021.708737)
Supplement: Supplementary Figure 1 — Bar graph comparing delta blood parameters lactate, pH and PCV (Hct) pre-, and post-ride in endurance and trotter exercise. [file Data_Sheet_1.pdf]

Supplementary Tables and Figures
MS T_c Endurance compared to Trotters

Supplementary Table S1. Table presenting T_c parameters in endurance (End) exercise (per 40 km) (H1-13) and trotters in warm-up (WU) and trotter (Tr) exercise (H14-25) and their recovery. T_c, gastrointestinal temperature; HR, heartrate; min, minutes; max, maximum; *italic* indicates extra 20 km exercise (n = 2 horses); delta (°C change), T_c change during exercise and recovery periods including first 10 min recovery period; Y/N, yes or no; NA, Not applicable; $^{\&}$ indicates recovery 40 min (H8 2nd 40 km) instead of 60 min in endurance horses (H1-8); – indicates no data collected/ not available; X indicates not reached T_c or HR; * indicates 38.5°C or higher; ** indicates 39°C or higher; ^ indicates increase of T_c during recovery; ^^ indicates max T_c during recovery; delta T_c, T_c change over time; *** indicates unstable (increase or decrease) over time; net AUC, area under the curve expressed as (°C x min); $^{\&\&}$ indicates short recovery of varying duration (3.5 – 7 min).

Horse number	Type exercise	Distance (km)	Duration exercise (min)	Duration recovery (min)	Baseline T_c (°C)	Max T_c (°C) exercise	Max T_c (°C) during recovery	Speed (km) at max T_c during exercise	HR (bpm) at max T_c during exercise or /recovery	Distance (km) at max T_c exercise	Time (min) to max T_c exercise	Time (min) to max T_c recovery	Time (min) max T_c to 38.5°C	Time (min) max T_c to 38.3°C	Time (min) max T_c to 38.0°C	Time (min) max T_c to base T_c	T_c (°C) end exercise	Delta T_c (°C) 40 km (total from base T_c)	T_c (°C) end recovery	Delta T_c (°C) recovery: from end exercise to max T_c during	T_c (°C) at 10 min post exercise	Delta T_c (°C) change 10 min^{-1} recovery	Delta T_c (°C) 10 min rec0very min^{-1}	Delta T_c (°C) end exercise to end recovery min^{-1}	Time (min) to HR < 60 bpm	Return to base T_c Y/N	Return to 38.0°C Y/N	Net AUC T_c (°C x min) during exercise (2nd 40 km only: net	Net AUC T_c (°C x min) recovery (recovery to HR 60 bpm)	Net AUC T_c from start 40 km (°C x min) min^{-1} duration exercise	Net AUC T_c (°C x min) min^{-1} duration recovery
1	End	40	165	60	37.7	39.2**	NA	11.5	150	17	82	-	- (Y)	-	-	X	39.1**	1.5	38.0	-1.2	-	-	-	0	14	N	N	85.3	-	0.5	-
1	End	2nd 40	182	60	38.0	39.5**	NA	17.2	91	31	167	-	14	48	X	X	39.3**	1.6 (1.9)	38.3	-1.0	38.7*	-0.5	-0.1	0	22	N	N	231.2 (176.7)	23.7	1.0	-0.4
2	End	40	236	60	37.8	38.8*	NA	15.9	133	21	126	-	NA	3	55	X	38.7*	1.1	37.9	-0.8	37.9	-1.2	-0.1	0	NA	N	Y	69.3	-	0.7	-
2	End	2nd 40	242	60	37.9	38.8*	NA	4.7	80	40	245	-	24	27	X	X	38.8*	0.8 (1.0)	38.3	-0.4	38.7*	0	0	0	16	N	N	135.2 (87.1)	-11.9	0.6	-0.2
3	End	40	207	60	37.8	38.6*	NA	20	132	39	192	-	57	77	44	57	38.1	0.7	37.8	-0.3	38.2	0	0	0	4	Y	Y	97.7	11.8	0.5	0.2
3	End	2nd 40	247	60	37.8	38.5*	NA	20	127	11	54	-	0	16	X	X	38.5*	0.7 (0.7)	38.1	-0.3	38.5*	+0.1	0	0	5	N	N	82.2 (82.2)	-17.4	0.3	-0.3
4	End	40	207	60	37.9	39.0**	NA	12	78	30	146	-	24	6	42	53	38.9*	1.1	37.9	-1.0	38.8*	-0.1	0	0	3	Y	Y	23.6	-33.1	0.8	-0.6
4	End	2nd 40	247	60	37.9	38.9*	NA	14	68	40	244	-	31	33	36	39	38.9*	1.0 (0.9)	37.8	-1.1	38.6*	-0.2	0	0	4	Y	Y	132.7 (38.6)	-43.9	0.5	-0.7
(4)	End	*Extra 20*	*144*	*(40)*	*37.8*	*38.8**	*NA*	*18*	*127*	*16*	*67*	-	*17*	*X*	*X*	*X*	*38.5**	*1.0 (0.9)*	*38.4*	*-0.1*	*38.6**	*0*	0	0	*11*	*N*	*N*	*90.6 (-70.8)*	*-1.5*	*(0.6)*	*(-0.04)*
5	End	40	186	60	38.3	39.3**	NA	21	63	40	181	-	55	X	X	X	39.2**	1.0	38.4	-0.8	39.2**	0	0	0	0	N	N	105.8	-22.3	0.6	-0.1
5	End	2nd 40	188	60	38.4	39.3**	NA	19	102	16	67	-	4	60***	X	106	38.6*	0.9 (1)	38.3	-0.3	38.2	-0.3	0	0	2	N	Y	92.1 (105.3)	-3.5	0.5	-0.2
6	End	40	179	60	37.7	38.6*	NA	13	114	12	54	-	NA	9	31	5	38.4	0.9	37.5	-0.9	37.8	-0.6	-0.1	0	5	Y	Y	107.7	-4.0	0.6	0.7
6	End	2nd 40	149	60	37.5	38.8*	NA	21	82	39	145	-	18	20	35	X	38.8*	1.2 (1.1)	37.7	-1.1	38.6*	-0.2	0	0	2	N	Y	110.5 (86.6)	-35.1	0.5	-0.6
6	End	*Extra 20*	*92*	*60*	*37.7*	*38.5**	*NA*	*17*	*89*	*20*	*150.5*	-	*3*	*22*	*X*	*X*	*38.5**	*0.8 (0.9)*	*38.0*	*-0.5*	*38.4*	*-0.1*	0	0	*0*	*N*	*N*	*34.3 (38.9)*	*-15.7*	*(0.4)*	*(0.3)*
7	End	40	148	60	37.6	39.2**	NA	N	152	-	129	-	11	13	14	X	38.9*	1.6	37.8	-0.8	38.6*	-0.3	0	0	5.5	N	Y	127.7	-55.0	0.9	-0.9
7	End	2nd 40	83	60	37.8	39.4**	39.91**	N	83	-	82	57	X >80	X	X	X	39.4**	2.1 (2.4)	39.6**	+0.2^	39.5**	+0.2	0	0	24***	N	N	62.5 (84.8)	18.5	0.8	0.2
8	End	40	209	60	37.8	39.0**	NA	16.5	120	40	148.5	-	7	60***	X***	X	39.0**	1.2	38.0	-0.9	38.8*	-0.2	0	0	11	N	N	122.3	-59.7	0.6	-0.7
8	End	2nd 40	187	40$^{\&}$	38.3	39.3**	NA	17.4	110	37	158	-	16	19	X	X	39.0**	1.0 (1.5)	38.2$^{\&}$	-0.8$^{\&}$	38.8*	-0.3	0	0	2	N	N	116.4 (215.8)	-22.1$^{\&}$	0.6	-0.6$^{\&}$
14	WU	1,54	4.12	3.39	38.3	38.3	38.3	NA	49	-	0	-	N/A	0	X	0	38.3	0	38.3	0	-	-	-	-	-	-	-	0	0	0	0
15	WU	1,54	4.09	2.55	37.0	37.8	38.0	NA	185	-	4	-	0	0	N/A	X	37.8	0.8	38.0	0	-	-	-	-	-	-	-	0	0.1	0	0
16	WU	1,54	4.12	3.5	37.5	37.8	37.9	NA	119	-	4	-	0	0	0	X	37.8	0.1	37.9	0	-	+0.1$^{\&}$	0	0	-	-	-	0.7	0.3	0	01
17	WU	1,54	4.14	3.5	38.1	38.2	38.4	NA	123/63	-	3	-	N/A	X	X	X	38.3	0.1	38.4	0	-	+0.1$^{\&}$	0	0	-	-	-	0.2	0.4	0.1	0.1
18	WU	1,54	4.23	5	37.5	37.5	-	NA	135/-	-	3	-	N/A	N/A	N/A	N/A	37.5	0	37.5	0	-	0	0	0	N/A	-	-	0.1	0	0	0
19	WU	1,54	3.51	4.5	38.	38.4	38.5	-	139/56	-	4	-	0	X	X	X	38.4	0.1	38.5	+0.1^	-	+0.2$^{\&}$	0	0	6	-	-	0.1	0.5 (0.1)	0	0.1
20	WU	1,54	3.52	5.1	37.1	37.8	38.0	NA	144/42	-	4	-	N/A	N/A	0	X	37.8	0.8	38.0	+0.1^	-	+0.1$^{\&}$	0	0	0.7	-	-	0.2	0.5 (0)	0.1	1.0
21	WU	1,54	3.46	6.9	38.0	38.0	38.1	NA	-/-	-	4	-	X	X	X	X	38.0	0	38.1	+0.1^	-	-0.1$^{\&}$	0	0	0.5	-	-	0.1	0.1 (0)	0	0
22	WU	1,54	3.41	6	37.7	37.8	38.0	NA	123/71	-	0	-	0	0	0	X	37.8	0.1	38.0	+0.2^	-	+0.2$^{\&}$	0	0	1	-	-	0.06	0	0	0
23	WU	1,54	3.38	6	38.0	38.0	38.0	NA	213/112	-	3	--	N/A	N/A	0	0	38.0	0	38.0	0^	-	0$^{\&}$	0	0	2.5	N	N	0	0	0.1	0
24	WU	1,54	3.46	5	37.7	37.8	NA	NA	128/-	-	2	-	N/A	N/A	0	0	37.8	0	37.8	+0.1^		-0.1$^{\&}$	0	0	0.8	N	N	0.1	0.1 (0)	0	0
25	WU	1,54	3.42	7	37.6	37.7	NA	NA	128/-	-	3	-	N/A	N/A	0	0	37.7	0.1	37.9	+0.1^	-	-0.1$^{\&}$	0	0	2.2	N	N	0.2	0.6 (0.1)	0.1	0.2
14	Tr	1,54	3.52	82	38.3	38.3^^	38.7	NA	142/142	-	4^^	65	N/A	0	-	-	38.3	0	38.4	+0.3^	38.3	0	0	0	9.5	N	N	0	6.4	0	0.8
15	Tr	1,54	3.51	14.5	38.4	38.6^^	39.0**	NA	152/72	-	12^^	7	X	-	-	-	38.6	0.9	38.9	+0.3^	39.0	+0.3	0	0	10	Y	Y	0.6	3.8	0.2	1.1
16	Tr	1,54	4.18	14	37.9	38.1^^	38.9*	NA	169/75	-	5^^	12	X	X	X	X	38.1	0.2	38.7	+0.9^	38.7	+0.6	0	0	X	N	N	0	5.0	0	0.4
17	Tr	1,54	4.14	15	38.4	38.5^^	38.9*	NA	139/67	-	4^^	6.5	X	X	X	X	38.5	0.1	38.8	+0.5^	38.9	+0.4	0	0	X	N	N	0.2	0.2	0.1	0.1
18	Tr	1,54	3.39	18	37.5	37.5^^	37.6	NA	NA/ 58	-	0^^	17	NA	NA	NA	NA	37.5	0	37.5	+0.1^	NA	NA	NA	0	5	N	N	-0.1	0.1	0	0
19	Tr	1,54	2.34	32	38.5	38.5^^	39.3**	-	84/73	-	2^^	22	X	X	X	X	38.5	0	39.3**	+0.4^	39.1	+0.6	+0.1	0	X	N	N	0	18.7	0	0.6
20	Tr	1,54	2.06	33.5	38.0	38.0^^	38.7*	NA	161/-		2^^	24	X	X	X	X	38.0	0	38.6	+0.7^	38.4	+0.4	0	0	27	N	N	1.2	16.1	0	0.5
21	Tr	1,54	2.06	83.5	38.1	38.2^^	39.1**	NA	80/-	--	2^^	72.5	X	X	X	X	38.2	0.1	39.1**	+0.9^	38.5	+0.3	0	0	X	N	N	0.5	44.1	0.2	0.5
22	Tr	1,54	2.07	15	38.0	38.1^^	38.7*	NA	149/-	-	3^^	14.5	X	X	X	X	38.1	0.1	38.7	+0.6^	38.5	+0.5	+0.1	+0.1	51	N	N	0.1	4.3	0	0.3
23	Tr	1,54	1.54	82	38.0	38.0^^	39.3**	NA	204/-=	-	2^^	79	X	X	X	X	38.0	0	39.3 **	+1.3^	38.3	+0.2	0	0	X	N	N	0	66.3	0	0.8
24	Tr	1,54	1.57	77	37.8	37.9^^	39.1**	NA	158/-	-	3^^	71	X	X	X	X	37.9	0.1	39.1**	+1.2^	38.2	+0.4	0	0	X	N	N	0	48.8	0	0.6
25	Tr	1,54	2.00	26	37.9	37.9^^	38.4	NA	154/-	-	2^^	20.5	NA	NA	X	X	37.9	0	38.3	+0.5^	38.2	+0.4	0	0	X	N	N	0	9.3	0	0.4

Supplementary Table S2. Table presenting Global positioning system (GPS), heartrate (HR) values and metabolic heat production (H, calculated in H1 – 13 only) in: endurance horses (H1-13) (End) per 40 km, and trotters (H14-25) during warm-up (WU) and trotter (Tr) exercise and recovery. Data are presented as mean ± SD. Min, minutes; km, kilometers; H, metabolic heat production expressed in kcal of kJ; VO_2 L^{-1}, oxygen per liters; COT, cost of transport, oxygen (ml) per kg bodyweight per meter (m) expressed in ml O_2 kg^{-1} m^{-1} exercise; – indicates no data collected/ not available/ not recorded; * indicates extra 20 km in italic; ** indicates speed only recorded during second 40 km; # indicates calculated values; X indicates HR 60 bpm was not reached at the time (min) of last recording.

Horse number	Type exercise	Distance (km)	Max speed (km h^{-1})	Mean speed (km h^{-1})	Mean HR (bpm)	Time to HR < 60 bpm (min)	Duration exercise (min)	Total H (kcal) exercise #	Total H (kJ) exercise #	Mean H (kcal) exercise min^{-1} #	Mean H (KJ) exercise min^{-1} #	Mean VO_2 (L O_2 min^{-1}) exercise #	Mean COT (ml O_2 kg^{-1} m^{-1}), exercise #
1	End	1st 40	26.1	13.8 ± 3.2	113 ± 31	14	165	12000	52000	110 ± 50 (10 -240)	450 ± 230 (30 – 1010)	19.8 ± 6.7 (1.4 – 30.5)	0.15 ± 0.09 (0.05 – 0.53)
1	End	2nd 40	26.1	15.3 ± 2.9	103 ± 15	22	182	22000	94000	110 ± 40 (20 – 240)	460 ± 170 (70 – 1000)	20.5 ± 6.3 (3.2 - 38.1)	0.13 ± 0.05 (0.05 – 0.37)
2	End	1st 40	22	12.9 ± 3.2	116 ± 21	NA	236	21000	87000	70 ± 40 (10 – 200)	300 ± 150 (30 – 850)	14.2 ± 6.8 (1.3 – 31.1)	0.15 ± 0.09 (0.05 – 0.53)
2	End	2nd 40	23.5	10.5 ± 4.3	110 ± 19	16	242	15000	63000	70 ± 40 (90 – 220)	290 ± 170 (90 – 930)	13.7 ± 7.5 (0.1 – 29.9)	0.13 ± 0.05 (0.05 – 0.38)
3	End	1st 40	28	12.8 ± 4.2	111 ± 24	4	207	14000	57000	60 ± 40 (0 – 220)	240 ± 170 (2 - 910)	11.2 ± 10.0 (0.1 – 30.0)	0.15 ± 0.22 (0.05 – 0.95)
3	End	2nd 40	30	13.6 ± 5.6	98 ± 23	5	247	19000	80000	70 ± 40 (0 – 210)	300 ± 180 (2 - 871)	13.7 ± 7.1 (0.1 – 29.9)	0.14 ± 0.08 (0.05 - 0.93)
4	End	1st 40	21	13.6 ± 3.3	110 ± 21	3	207	18000	740000	60 ± 30 (10 – 120)	260 ± 120 (40 – 500)	15.6 ± 6.8 (2.3 – 29.9)	0.14 ± 0.08 (0.05 – 0.89)
4	End	2nd 40	22	12.6 ± 4.4	114 ± 36	4	247	21000	88000	60 ±30 (0 – 120)	240 ± 120 (0 – 500)	13.5 ± 7.0 (0.1 – 29.6)	0.14 ± 0.07 (0.05 – 0.81)
_4_	_End_	_20*_		_10.4 ± 4.4_	_106 ± 27_	_11_	_144_	_10000_	_40000_	_20 ± 120 (0 – 70)_	_70 ± 04 (0 – 270)_	_12.1 ± 6.9 (0.2 – 29.9)_	_0.13 ± 0.08 (0.05 – 0.94)_
5	End	1st 40	24	16.6 ± 2.7	108 ± 21	0	186	18000	74000	80 ± 40 (20 – 340)	340 ± 170 (80 – 140)	15.4 ± 6.6 (3.9 – 30)	0.13 ± 0.07 (0.047 – 0.687)
5	End	2nd 40	27	15.3 ± 5.2	110 ± 26	2	188	17000	71000	70 ± 50 (0 – 270)	310 ± 190 (0 – 1120)	13.7 ± 7.1 (0.1 – 29.8)	0.131 ± 0.069 (0.05 – 0.70)
6	End	1st 40	24	16.3 ± 3.2	91 ± 21	5	179	14000	60000	90 ± 50 (20 – 230)	390 ± 190 (100 – 980)	17.3 ± 6.8 (4.7 – 30.0)	0.11 ± 0.07 (0.05 - 0.69)
6	End	2nd 40	25	17.4 ± 3.5	89 ± 22	2	149	13000	55000	80 ± 60 (0 – 270)	350 ± 240 (0 – 1140)	14.5 ± 7.7 (0.1 – 29.3)	0.13 ± 0.07 (0.05 – 0.70)
_6_	_End_	_20*_	_23_	_15.8 ± 2.4_	_99 ± 21_	_0_	_92_	_8000_	_33000_	_30 ± 40 (0 – 210)_	_110 ± 150 02 – 900)_	_5.3 ± 6.9 (0.1 – 29.5)_	_0.13 ± 0.04 (0.04 – 0.46)_
7	End	1st 40	-	-	123 ± 24	5.5	148	-		-	-	-	-
7	End	2nd 40	-	-	122 ± 9	24	83	-		-	-	-	-
8	End	1st 40	21	14.3 ± 4.8	123 ± 30	11	209	15000	64000	70 ± 30 (0 – 180)	300 ± 1230 (10 – 760)	14.0 ± 5.5 (0.4 – 29.9)	0.13 ± 0.07 (0.05 – 0.91)
8	End	2nd 40	23.5	16.2 ± 3.2	130 ± 29	2	187	18000	74000	80 ± 30 (20 – 210)	340 ± 130 (70 – 870)	15.8 ± 5.1 (3.1 – 29.9)	0.13 ± 0.05 (0.05 – 0.90)
9	End	1st 40	21.1	14.4 ± 2.1	107 ± 17	2	219	19000	80000	80 ± 29 (10 – 240)	360 ± 120 (50 – 990)	16.5 ± 4.7 (2.2 – 297)	0.13 ± 0.05 (0.05 – 0.91)
9	End	2nd 40	22.4	13.6 ± 3.8	109 ± 18	5	224	20000	84000	80 ± 40 (0 – 220)	340 ± 150 (10 – 930)	15.6 ± 6.1 (0.5 – 44.3)	0.14 ± 0.08 (0.05 – 0.90)
10	End	1st 40	21.1	13.3 ± 3.6	106 ± 24	2	215	14000	60000	70 ± 30 (10 – 180)	280 ± 110 (30 – 750)	13.2 ± 4.9 (0.1 – 0.1)	0.12 ± 0.04 (0.05 – 0.81)
10	End	2nd 40	23.8	11.4 ± 5.8	107 ± 27	1.5	215	13000	53000	60 ± 330 (0 -160)	260 ± 140 (10 – 660)	12.2 ± 6.5 (0.3 – 28.7)	0.13 ± 0.04 (0.04 – 0.54)
11	End	1st 40	19.2	12.2 ± 4.3	123 ± 46	0	218	15000	64000	60 ± 30 (0 – 210)	260 ± 130 (0 – 880)	12.2 ± 5.5 (0.1 – 30.0)	0.13 ± 0.05 (0.05 – 0.41)
12	End	2nd 40	20	14.3 ± 3.7	124 ± 26	5	185	17000	71000	80 ± 30 (0 – 220)	330 ± 140 (10 – 9220)	15.3 ± 0.5 (0.5 – 29.8)	0.13 ± 0.05 (0.05 - 0.59)
12	End	40	21	14.9 ± 2.1	139 ± 34	6	209	20000	85000	90 ± 30 (10 – 230)	380 ± 130 (20 – 950)	17.5 ± 4.5 (1.1 – 29.9)	0.13 ± 0.05 (0.05 – 0.64)
13	End	1st 40	-	-	143 ± 32	2	184	-		-	-	-	-
13	End	2nd 40	22	15.0 ± 4.2	112 ± 28	5	206	19000**	78000	80 ± 30 (0 – 30)	320 ± 100 (10 – 820)	15.2 ± 4.4 (0.5 – 29.8)	0.13 ± 0.06 (0.05 – 0.90)
14	WU	1540	36.9	30.4 ± 6.3	163 ± 36	3	4.1						
15	WU	1540	39.8	30.9 ± 5.6	165 ± 32	3	4.1						
16	WU	1540	42.3	31.9 ± 7.0	116 ± 20	3	4.1						
17	WU	1540	40.0	32.1 ± 6.2	116 ± 21	3	4.1						
18	WU	1540	38.4	37.1± 6.7	153 ± 22	3	4.2						
19	WU	1540	-	-	119 ± 25	6 (38.4)	3.5						
20	WU	1540	25	22.2 ± 2.9	126 ± 13	48 s (37.8)	3.5						
21	WU	1540	25	23.4 ± 2.1	-	-	3.5						
22	WU	1540	26.3	23.5 ± 2.9	145 ± 12	1 (37.8)	3.4						
23	WU	1540	28.1	23.3 ± 2.9	177 ± 24	2.5 (38.0)	3.4						
24	WU	1540	25.2	22.5 ± 2.5	104 ±77	1	3.5						

25	WU	1540	24.7	22.9 ± 2.4	140 ± 10	2.5	3.4
14	Tr	1540	51.3	35.5 ± 7.9	167 ± 25	9.5 (38.8)	3.5
15	Tr	1540	42	35.3 ± 6.7	168 ± 23	X (126min:HR126; 38.9)	3.5
16	Tr	1540	52.5	41.4 ± 9.5	130 ± 26	X (14min HR70)	4.2
17	Tr	1540	52.3	42.2 ± 8.5	131 ± 25	X (15min: HR65; 38.8)	4.1
18	Tr	1540	47.1	37.1 ±6.7	153 ± 22	4 (37.6)	3.4
19	Tr	1540	-	-	161 ± 55	-	2.3
20	Tr	1540	49.1	39.2 ± 9.2	144 ± 33	27 [38.6]	2.1
21	Tr	1540	54	41.5 ± 8.6	-	-	2.1
22	Tr	1540	47.9	39.8 ± 8.9	137 ± 25	51 [38.7]	2.1
23	Tr	1540	52.7	43.8 ± 8.8	169 ± 28	X (12min: HR115; 38.8)	1.5
24	Tr	1540	51.8	42.4 ±9.8	129 ± 17	X (12min: HR101; 38.2)	1.6
25	Tr	1540	51.8	41.5 ± 8.9	129 ± 21	X (8.5min: HR94; 38.2)	2.0

Supplementary Table S3. Table presenting blood parameters of endurance horses and trotters (only lactate, PCV, pH) pre- and post-ride. Data are presented as mean ± SD and in addition, delta (difference with previous value) presented between brackets; *n* =, indicates number of samples; * indicates number of sampled endurance horses; PCV, packed cell volume (Hct); TS, total solids; TP, total protein; PCO_2, partial pressure of carbon dioxide; PO_2, partial pressure of oxygen; HCO_3^-, bicarbonate; BE, base excess; SO_2, oxygen saturation; Ca Tot, total calcium; tCO_2, total CO_2; CK, creatinine kinase; AST, aspartate amino transferase; BUN, blood urea nitrogen; Creat, creatinine; Tbil, total bilirubin; Alb, albumin; Glob, globulin; WBC, white blood cell count in horses 7,8,12,13; Lymp, lymphocyte count; Neutr, neutrophil count.

Rest endurance exercise*	Post 40 km (*n* = 13*)	Post 80 km, *n* = 11*	Post 100 km, *n* = 2*	Rest warm-up exercise, *n* = 12	Post warm-up exercise, *n* = 12	Post trotter exercise, *n* = 12	
1.0 ± 0.3	1.2 ± 0.6 (0.2)	1.5 ± 0.4 (0.2)	1.3 ± 0.9 (-0.2)	1.0 ± 0.5	1.6 ± 1.0 (0.3)	9.4 ± 4.0 (8.0)	**Lactate (mmol L^{-1}, *n* = 13*)**
42.5 ± 11.3	46.6 ± 4.8 (4.2)	48.6 ± 5.7 (2.0)	48.5 ± 0.7 (-0.1)	41.5 ± 5.3	47.5 ± 2.5 (3.3)	53.1 ± 2.5 (5.2)	**PCV (%, *n* = 13*)**
7.4 ± 0	7.4 ± 0 (0)	7.4 ± 0 (0)	7.4 ± 0 (0)	7.3 ± 0.1	7.3 ± 0.1 (0)	7.2 ± 0.2 (-0.1)	**pH (*n* = 9*)**
6.0 ± 0.8	6.4 ± 1.0 (0.4)	7.0 ± 0.8 (0.6)	5.8 ± 0.2 (-1.3)				**TP/TS (g dL^{-1}, *n* = 13*)**
49.4 ± 3.8	48.2 ± 3.1 (-1.0)	46.5 ± 4.3 (-1.6)	50.0 ± 2.7 (3.5)				**PCO_2 (mmHg, *n* = 9*)**
32.0 ± 6.2	31.1 ± 3.8 (-0.9)	32.6 ± 6.7 (1.55)	27.3 ± 6.2 (-5.3)				**PO_2 (mmHg, *n* = 9*)**
29.4 ± 2.3	31.6 ± 1.9 (2.1)	30.4 ± 2.9 (-1.2)	32.4 ± 0.4 (1.9)				**HCO_3^- (mmol L^{-1}, *n* = 9*)**
4.36 ± 2.41	7.19 ± 2.21 (2.8)	6.10 ± 3.06 (-1.09)	7.9 ± 0 (2.8)				**BE (mmol L^{-1}, *n* = 9)**
62.6 ± 18.1	61.1 ± 13.7 (-1.4)	62.2 ± 13.7 (1.1)	50.3 ± 13.7 (-11.9)				**SO_2 (%, *n* = 9*)**
136.6 ± 2.3	138.3 ± 2.7 (1.8)	135.6 ± 3.2 (-2.7)	135.5 ± 2.1 (-0.1)				**Na^+ (mmol L^{-1}, *n* = 13*)**
3.7 ± 0.3	3.6 ± 0.3 (-1.6)	3.5 ± 0.4 (-0.1)	3.3 ± 0 (-0.2)				**K^+ (mmol L^{-1}, *n* = 13*)**
1.3 ± 0.1	1.2 ± 0.1 (0)	1.2 ± 0.1 (0)	1.2 ± 0.2 (0)				**Ca^{++} (mmol L^{-1}, *n* = 9*)**
3.2 ± 0.2	3.2 ± 0.2 (0)	3.1 ± 0.1 (-0.1)					**Ca Tot (mmol L^{-1}, *n* = 4*)**
30.3 ± 1.8	32.0 ± 2.4 (1.7)	29.5 ± 4.8 (-2.5)	33.9 ± 0.4 (4.4)				**TCO_2, (*n* = 13*)**
6.3 ± 1.7	5.9 ± 0.8 (-0.5)	4.6 ± 1.0 (-1.3)	3.8 ± 1.6 (-0.8)				**Glucose (mmol L^{-1}, *n* = 13*)**
367 ± 13	563 ± 416 (196)	2586 ± 3395 (2023)					**CK (IU L^{-1} (*n* = 4*)**
549 ± 271	444 ± 247 (-105)	580 ± 377 (136)					**AST (IU L^{-1} (*n* = 4*)**
19.0 ± 0	18.8 ± 0.5 (0)	21.7 ± 1.2 (2.9)					**BUN (mg dL^{-1}, *n* = 4*)**
0.8 ± 0.1	1.1 ± 0.2 (0.3)	1.2 ± 0.3 (1.5)					**Creat (mg dL^{-1}, *n* = 4*)**
1.9 ± 0.1	2.0 ± 0.5 (0.1)	2.4 ± 0.6 (0.5)					**Tbil (mg dL^{-1}, *n* = 4)**
3.5 ± 0	3.4 ± 0.3 (-0.1)	3.4 ± 0.4 (0)					**Alb (g dL^{-1}, *n* = 4*)**
3.2 ± 0.7	4.4 ± 1.5 (1.2)	3.3 ± 0.1 (-1.1)					**Glob (g dL^{-1}, *n* = 4*)**
4.7 ± 1.7	5.9 ± 1.3 (1.2)	6.5 ± 2.4 (0.6)					**WBC (10^9 L^{-1}, *n* = 4*)**
0.8 ± 0.2	0.6 ± 0.3 (-0.2)	0.6 ± 0.1 (0)					**Lymph (10^9 L^{-1}, *n* = 4*)**
2.1 ± 0.2	5.2 ± 2.3 (3.1)	5.8 ± 2.4 (0.6)					**Neutr (10^9 L^{-1} (*n* = 4*)**

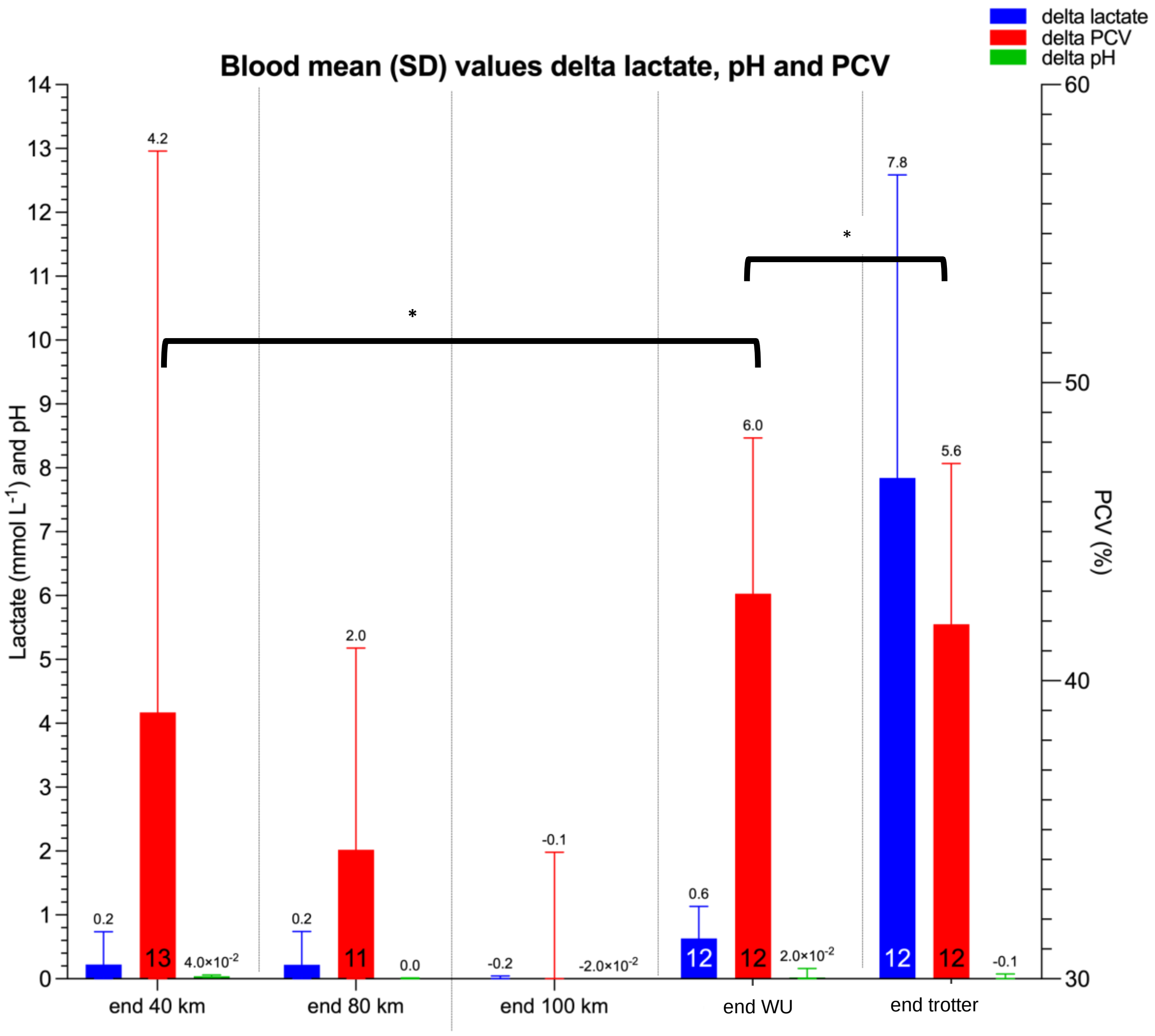

Supplementary Figure S1. Graph comparing delta (pre- and post-ride) blood parameters lactate, pH and PCV (Hct) at end 40, 80, 100 km endurance exercise, end warm-up (WU) and end trotter exercise (both during 1540 m). * indicates a significant difference in post-exercise delta lactate value between endurance, trotter WU and trotter exercise ($p < 0.0001$) while the delta PCV (Hct) and pH did not reveal any significant difference between the types of exercise ($p = 0.23$ and $p = 0.34$). Mean values less than 0.1 are not displayed in the graph.
